# Distributed intelligence in industrial and automotive cyber–physical systems: a review

**DOI:** 10.3389/frobt.2024.1430740

**Published:** 2024-10-28

**Authors:** Nikos Piperigkos, Alexandros Gkillas, Gerasimos Arvanitis, Stavros Nousias, Aris Lalos, Apostolos Fournaris, Panagiotis Radoglou-Grammatikis, Panagiotis Sarigiannidis, Konstantinos Moustakas

**Affiliations:** ^1^ Industrial Systems Institute, Athena Research Center, Patras, Greece; ^2^ Department of Electrical and Computer Engineering, University of Patras, Patras, Greece; ^3^ Chair of Computational Modeling and Simulation, School of Engineering and Design, Technical University of Munich, Munich, Germany; ^4^ Department of Electrical and Computer Engineering, University of Western Macedonia, Kozani, Greece; ^5^ K3Y Ltd., Sofia, Bulgaria

**Keywords:** cyber–physical systems, cyber–physical system of systems, cyber–physical–social system, human-in-the-loop, human-machine interfaces

## Abstract

Cyber–physical systems (CPSs) are evolving from individual systems to collectives of systems that collaborate to achieve highly complex goals, realizing a cyber–physical system of systems (CPSoSs) approach. They are heterogeneous systems comprising various autonomous CPSs, each with unique performance capabilities, priorities, and pursued goals. In practice, there are significant challenges in the applicability and usability of CPSoSs that need to be addressed. The decentralization of CPSoSs assigns tasks to individual CPSs within the system of systems. All CPSs should harmonically pursue system-based achievements and collaborate to make system-of-system-based decisions and implement the CPSoS functionality. The automotive domain is transitioning to the system of systems approach, aiming to provide a series of emergent functionalities like traffic management, collaborative car fleet management, or large-scale automotive adaptation to the physical environment, thus providing significant environmental benefits and achieving significant societal impact. Similarly, large infrastructure domains are evolving into global, highly integrated cyber–physical systems of systems, covering all parts of the value chain. This survey provides a comprehensive review of current best practices in connected cyber–physical systems and investigates a dual-layer architecture entailing perception and behavioral components. The presented perception layer entails object detection, cooperative scene analysis, cooperative localization and path planning, and human-centric perception. The behavioral layer focuses on human-in-the-loop (HITL)-centric decision making and control, where the output of the perception layer assists the human operator in making decisions while monitoring the operator’s state. Finally, an extended overview of digital twin (DT) paradigms is provided so as to simulate, realize, and optimize large-scale CPSoS ecosystems.

## 1 Introduction

In the past few years, there has been a significant investment in cyber–physical systems of systems (CPSoSs) in various domains, like automotive, industrial manufacturing, railways, aerospace, smart buildings, logistics, energy, and industrial processes, all of which have a significant impact on the economy and society. The automotive domain is transitioning to the system of systems approach, aiming to provide a series of emergent functionalities like traffic management, collaborative car fleet management, or large-scale automotive adaptation to the physical environment, thus providing significant environmental benefits (e.g., air pollution reduction) and achieving significant societal impact.

Similarly, large infrastructure domains, like industrial manufacturing (Nota et al., 2020), are evolving into global, highly integrated CPSoSs that go beyond pure production and cover all parts of the value chain, including research, design, and service provision. This novel approach can enable a high level of flexibility, allowing for rapid adaptation to customer requirements, a high degree of product customization, and improved industrial sustainability. Furthermore, achieving collective behavior in CPSoS-based solutions for large-scale control processes will help citizens improve their quality of life through smart, safe, and secure cities, energy-efficient buildings, and green infrastructures (i.e., lighting, water, and waste management), as well as smart devices and services for smart home functionality, home monitoring, health services, and assisted living.

However, in practice, there are significant challenges in the applicability and usability of CPSoSs that need to be addressed to take full advantage of the CPSoS benefits and sustain/extend their growth. The fact that even a small CPSoS (e.g., a connected car) consists of several subsystems and executes thousands of lines of code highlights the complexity of the system-of-systems solution and the extremely elaborate CPSoS orchestration required, highlighting the need for an approach beyond traditional control and management centers (Engell et al., 2015). Given this complexity, having a centralized authority that handles all CPSoS processes, subsystems, and control loops seems to be challenging to capture and implement, thus pointing to the need for a different design, control, and management approach. The decentralization of CPSoS processes and overall functionality by assigning tasks to individual cyber–physical systems (CPSs) within the system of systems can be a reasonable solution. However, the collaborative mechanisms between CPSs (that constitute the CPSoS behavior) remain a point of research since appropriate tools and methodologies are needed to ensure that the expected system-of-systems functional requirements are met (the CPSoS operates as it should be) and that non-functional requirements are fulfilled (the CPSoS remains resilient, safe, and efficient). Another critical challenge in effectively developing CPSoSs is the need for integrating social and human factors into the design process of CPSs so that the cyber, physical, and human layers can be efficiently coordinated and operated (Zhou et al., 2020). Compared with traditional CPSs, cyber–physical–social systems (CPSSs) regard humans as an important factor of the system and, therefore, incorporate human-in-the-loop (HITL) mechanisms into system operations so as to increase the trustworthiness of the overall CPSoS. To be more concise, creating an intelligent CPSoS environment relies on both modern technology and the natural resources provided by its inhabitants. Specifically, both “things” (objects and devices) and “humans” are essential for making smart environments even smarter. People benefit from smart services made possible by today’s technology while simultaneously contributing to the enhancement of business intelligence. In this context, CPSS, as the human-in-the-loop counterpart of CPSs, can be used to gather information from humans and provide services with user awareness, creating a more responsive and personalized intelligent environment.

To address the complex challenges in CPSoSs, researchers have proposed a two-layer architecture consisting of a perception layer and a behavioral layer. This approach serves as a foundation for advancing CPSoS research and development across multiple critical areas. The proposed architecture aims to enhance functionality, reliability, adaptability, and the overall situational awareness (Chen and Barnes, 2014) of CPSoSs in various domains, from automotive and industrial manufacturing to smart cities and healthcare. Situational awareness refers to the collective understanding of an environment shared among multiple agents or entities—whether human or machine—who work together to achieve a common goal. More specifically, this concept of collective understanding emphasizes that situational awareness is not confined to individual knowledge but is distributed across team members. By cooperating, participants can better understand complex environments, adapt to dynamic changes, and respond more efficiently to evolving situations. As such, by focusing on these two interconnected layers, researchers can tackle the intricate issues of system integration, human–machine interaction, and real-time decision-making that are essential for the next generation of CPSoSs. The perception layer focuses on enhancing situational awareness through sophisticated algorithms for object detection, cooperative scene analysis, cooperative localization, and path planning. Research in this layer aims to develop effective perception mechanisms that are foundational for achieving higher levels of autonomy and reliability in CPSoSs. These advancements will enable CPSoSs to interact more intelligently with their environment and make informed decisions based on comprehensive situational awareness. Additionally, the behavioral layer focuses on integrating human operators into CPSoSs, recognizing the crucial role of human knowledge, senses, and expertise in ensuring operational excellence. This layer introduces the HITL approach, which allows continuous interaction between humans and CPSoS control loops. It addresses applications in which humans directly control CPSoS functionality, systems passively monitor human actions and biometrics, and hybrid combinations of both. The behavioral layer explores advanced Human-Machine Interfaces (HMI), including speech recognition, gesture recognition, and extended reality technologies, to enhance situational awareness and enable seamless human–system interaction. Furthermore, it investigates the prediction of operator intentions to improve collaboration between humans and CPSoSs, particularly in industrial scenarios where safety and efficiency are paramount. By integrating human expertise and intuition alongside automated processes, this layer aims to create a true human–machine symbiosis, vital for maintaining system flexibility and responsiveness in dynamic environments and unforeseen events. Key research directions within this two-layer framework include decentralized control and management, human-in-the-loop integration, data analytics and cognitive computing, real-time processing and decision making, and collaborative mechanisms between individual CPSs. These areas of study aim to develop more intelligent, responsive, and human-centric systems that can adapt to the complex demands of our interconnected world.

In addition to these approaches, digital twins (DTs) are an emerging technology that assists in addressing the challenges within CPSoSs (Mylonas et al., 2021). DTs create accurate virtual replicas of physical systems, allowing for continuous observation of system performance, real-time data integration, and predictive maintenance, thus improving the reliability and efficiency of CPSoSs (Tao et al., 2019). DTs facilitate better decision-making by providing a comprehensive view of the entire system and enabling the simulation of various scenarios for proactive planning and response (Wang et al., 2023b). Furthermore, the integration of multiple CPSoSs through DTs can significantly enhance task performance. By enabling seamless communication and coordination among different CPSoSs, DTs ensure that each subsystem can efficiently share data and resources, leading to improved overall system performance. For example, in smart city environments, integrating transportation systems, energy grids, and public safety networks through DTs can optimize urban operations, reduce response times in emergencies, and enhance the overall quality of life for residents (Jafari et al., 2023). By integrating DTs into the CPSoS framework, systems can achieve higher efficiency, reliability, and adaptability. This synergy between DTs and CPSoSs leads to smarter, more resilient, and more efficient systems across various domains, providing robust solutions to complex challenges and contributing to the overall improvement of system performance and human wellbeing.

The remainder of this survey is organized as follows. In Section 2, we present the related work and outline the contributions of this study. Section 3 describes the conceptual architecture of CPSs. Section 4 delves into the perception layer, summarizing relevant works and providing detailed insights. Section 5 focuses on the behavioral layer, offering a comprehensive summary of pertinent research. Section 6 discusses the role of digital twins in optimizing the CPSoS ecosystem. Section 7 identifies open research questions, while Section 8 highlights key lessons learned. In Section 9, we provide a detailed discussion of various aspects of the study. Finally, Section 10 concludes the survey.

## 2 Related work and contribution

Many of the recent review papers discuss how the CPSs are utilized in emerging applications. Chen (2017) conducted an extensive literature review on CPS applications. Sadiku et al. (2017) provided a brief introduction to CPSs, their applications, and challenges. Yilma et al. (2021) presented the SoA perspectives on CPSs regarding definitions, underlining principles, and application areas. Other survey papers focus more on very specific areas. Haque et al. (2014) presented a survey of CPS in healthcare applications, characterizing and classifying different components and methods that are required for the application of CPS in healthcare, while Oliveira et al. (2021) presented the use of CPSs in the chemical industry. On the other hand, architecture and CPS characteristics are also common issues that are discussed in many survey papers. Hu et al. (2012) reviewed previous works of CPS architecture and introduced the main challenges, which are real-time control, security assurance, and integration mechanisms. CPS characteristics (like generic architecture, design principles, modeling, dependability, and implementation) and their application domains are also presented by Liu and Wang (2020). Lozano and Vijayan (2020) presented the current state-of-the-art, intrinsic features (like autonomy, stability, robustness, efficiency, scalability, safe trustworthiness, reliable consistency, and accurate high precision), design methodologies, applications, and challenges for CPS. Liu et al. (2017b) discussed the development of CPS from the perspectives of the system model, information processing technology, and software design. Oliveira et al. (2021) discussed the use of artificial intelligence to confer cognition to the system. Topics such as control and optimization architectures and digital twins are presented as components of the CPSs. Al-Mhiqani et al. (2018) investigated the current threats on CPSs (e.g., the type of attack, impact, intention, and incident categories). Leitão et al. (2022) provided an analysis of the main aspects, challenges, and research opportunities to be considered for implementing collective intelligence in industrial CPSs. Estrada-Jimenez et al. (2023) explored the concept of smart manufacturing, focusing on self-organization and its potential to manage the complexity and dynamism of manufacturing environments. It presents a systematic literature review to summarize current technologies, implementation strategies, and future research directions in the field. Pulikottil et al. (2023) explored the integration of multi-agent systems in cyber–physical production systems for smart manufacturing, offering a thorough review and SWOT analysis validated by industry experts to assess their potential benefits and challenges. Hamzah et al. (2023) provided a comprehensive overview of CPSs across 14 critical domains, including transportation, healthcare, and manufacturing, highlighting their integration into modern society and their role in advancing the fourth industrial revolution. Additionally, DT-based survey works focus on realizing automotive CPS (Xie et al., 2022a), achieving high adaptability with a short development cycle, low complexity, and high scalability, which meet various design requirements during the development process, how the interconnection between different components in CPSs and DTs affect the smart manufacturing domain (Tao et al., 2019), as well as presenting the potential of DTs as a means to reinforce and secure CPSs and Industry 4.0 in general (Lampropoulos and Siakas, 2023).

In this survey, we focus on the CPS architecture and its modules that are used to increase the situational awareness of the CPSoS users. Considering the importance of human integration in CPSs, we include the HITL component and human–machine interaction to realize the CPSS paradigm. Additionally, we emphasize the critical role of DTs in optimizing CPSoS ecosystems. The main contributions of this paper can be summarized as follows:

• Comprehensive review of current best practices in connected CPSs.

• Investigation of a dual-layer architecture encompassing a perception layer and a behavioral layer, where the perception layer focuses on enhancing situational awareness and the behavioral layer integrates human operators through HITL mechanisms and advanced HMI technologies.

• Presentation of different datasets and sources of data available to the research community. Perception algorithms related to scene understanding (object detection and tracking, pose estimation), localization mapping, and path planning are thoroughly investigated. The behavioral part focuses on decision making and human-in-the-loop control.

• Discussion on the integration of DTs into CPSoSs, highlighting their applications in smart cities, intelligent transportation systems, and aerial traffic monitoring.

## 3 Conceptual architecture

### 3.1 CPSoSs are heterogeneous systems

They consist of various autonomous CPSs, each with unique performance capabilities, criticality levels, priorities, and pursued goals. CPSs, in general, are self-organized and, on several occasions, may have conflicting goals, thus competing to get access to common resources. However, from a CPSoS perspective, all CPSs must also harmonically pursue system-based achievements and collaborate to make system-of-system-based decisions and implement the CPSoS behavior. Considering that CPSoSs consists of many CPSs, finding the methodology to achieve such an equilibrium in a decentralized way is not an easy task. The above issue becomes more complex when we also consider the amount of data to be exchanged between CPSs and the processing of those data. The collection of data and the data analytics need to be refined in such a way that only the important information is extracted and forwarded to other CPSs and the overall system. Furthermore, mechanisms to handle large amounts of data in a distributed way are needed to extract cognitive patterns and detect abnormalities. Thus, local data classification, labeling, and refinement mechanisms should be implemented in each CPS to offload the complexity and communication overhead at the system-of-systems level (Atat et al., 2018).

In the above-described setup, we cannot overlook the fact that CPSoSs depend on humans since humans are part of the CPSoS functionality and services, interact with the CPSs, and contribute to the CPSoS behavior. Operators and managers play a key role in the operation of CPSoSs and make many important decisions, while in several cases, human CPS users are the key players in the CPSoS main role (thus forming cyber–physical human systems). Thus, we need to structure a close symbiosis between computer-based systems and human operators/users and constantly enhance human situational awareness as well as devise a collaborative mechanism for handling CPSoS decisions, forcing the CPSoSs to comply with human guidelines and reactions. Novel approaches to human–machine interfaces that employ eXtended Reality (XR) principles need to be devised to help humans gain fast and easy-to-grasp insights into CPSoS processes while also enabling their seamless integration into CPSoS operations.

**FIGURE 1:**
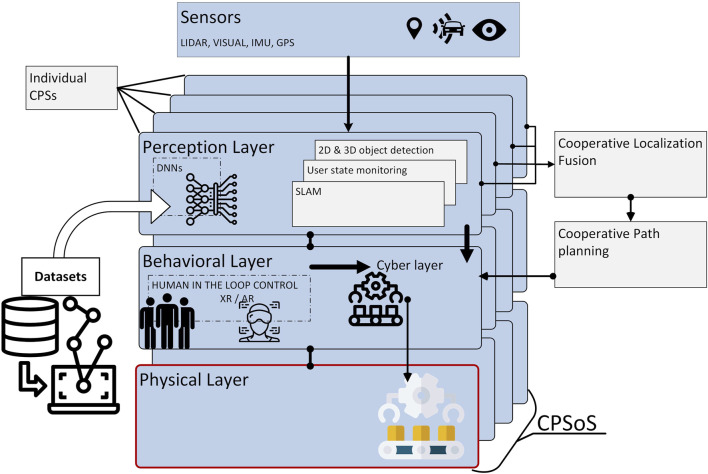
Cognitive cyber–physical system of system architecture with human-in-the-loop control.

As shown in Figure 1, it is assumed that a CPSoS consists of interconnected CPSs, each acting independently while also collectively functioning as part of the CPSoS. We also assume that each individual CPS is composed of a *perception module* and a *behavioral or decision-making module* while bearing actuation capacities represented by the *physical layer*; this configuration facilitates the coordination with the other connected CPSs toward the collective implementation of a common goal or mission. A key aspect of the proposed architecture is the integration of various sensors, including redundant, complimentary, and cooperative sensors across nodes. More specifically, incorporating redundant sensors in interconnected CPSs is necessary to improve reliability, fault tolerance, and system safety. Redundant sensors provide backup in the event of failure of primary sensors, ensuring continued operation without disruption and maintaining the integrity and reliability of CPS (Bhattacharya et al., 2023). Additionally, the use of complementary sensors in heterogeneous systems allows for cross-verification of data, better coverage of sensor limitations, and improved decision-making in dynamic environments (Alsamhi et al., 2024). For example, combining visuals with depth sensors in autonomous vehicles helps enhance object detection, environmental mapping, and path planning. The diverse data from complementary sensors can be fused to produce more accurate and comprehensive situational awareness. Finally, cooperative sensors on individual CPSs work together to improve the accuracy and robustness of measurements and operations. These sensors share information and collaborate to address limitations inherent in individual sensors. For instance, in robotics, multiple sensors like cameras, inertial measurement units (IMUs), and proximity sensors can cooperate to provide accurate localization and object detection (Zhang and Singh, 2015).

The aggregation of the individual perception modules formulates the perception layer of the CPSoS, while the sensor, behavioral, and physical layers represent the summation of sensing, decision making, and actuation capabilities of the CPSoS. The perception layer can also be envisioned as a cognitive engine that employs appropriate algorithmic approaches for effective scene understanding, a task predominately accomplished today by deep neural network architectures. To this end, data collection and annotation are crucial for the training of AI models to undertake such tasks. This review paper sheds light upon all of the aforementioned aspects of interconnected CPSs.

## 4 Perception layer

### 4.1 Cooperative scene analysis

#### 4.1.1 Background on object detection from 2D and 3D data

Object detection has evolved considerably since the appearance of deep convolutional neural networks (Zhao et al., 2019b). Nowadays, there are two main branches of proposed techniques, namely, two-stage and single-stage detectors.

In the first one, the object detectors, using two stages, generate region proposals, which are subsequently classified into the categories that are determined by the application at hand (e.g., vehicles, cyclists and pedestrians, in the case of autonomous driving). Some important, representative, high-performance examples of this first branch are R-CNN (Girshick et al., 2014), Fast R-CNN (Girshick, 2015), Faster R-CNN (Ren et al., 2016), spatial pyramid pooling net (He et al., 2015), region-based fully convolutional network (R-FCN) (Dai et al., 2016), feature pyramid network (FPN) (Lin et al., 2017), and mask R-CNN (He et al., 2017). In the second branch, object detection is cast to a single-stage, regression-like task with the aim to provide directly both the locations and the categories of the detected objects. Notable examples, here, are Single Shot MultiBox Detector (SSD) (Liu et al., 2016), SqueezeDet (Wu et al., 2017),YOLO (Redmon et al., 2016), YOLOv3 (Redmon and Farhadi, 2018) and EfficientDet (Tan et al., 2020). A recent review on object detection using deep learning (Zhao et al., 2019b) provides inquisitive insight into the aforementioned approaches to object detection.

Object detection in LiDAR point clouds is a three-dimensional problem where the sampled points are not uniformly distributed over the objects in the scene and do not directly correspond to a Cartesian grid. Three-dimensional object detection is dominantly performed using 3D convolutional networks due to the irregularity and lack of apparent structure in the point cloud. Several transformations take place to match the point cloud to feature maps that are forwarded into deep networks. Commendable detection outcomes have appeared in the literature as early as 2016. Li et al. (2016a) projected the 3D points onto a 2D map and employed 2D fully convolutional networks to successfully detect cars in a LiDAR point cloud, reaching an accuracy of 71.0% for car detection of moderate difficulty. A follow-up paper by Li (2017) proposed 3D fully convolutional networks, reporting an accuracy of 75.3% for car detection of moderate difficulty. However, since dense 3D fully convolutional networks demonstrate high execution times, Yan et al. (2018) investigated an improved sparse convolution method for such networks, which significantly increases the speed of both training and inference. According to KITTI benchmarks, the reported accuracy reaches 78.6% for car detection of moderate difficulty. To revisit 2D convolutions in 3D object detection, Lang et al. (2019) proposed a novel encoder called PointPillars that utilizes PointNets to learn a representation of point clouds organized in vertical columns (pillars) and subsequently employs a series of 2D convolutions. PointPillars reported an accuracy of 77.28% in the same category. Shi et al. (2019) proposed PointRCNN for 3D object detection from raw point clouds. They devised a two-stage approach where the first stage generates bottom-up 3D proposals and the second stage refines these proposals in the canonical coordinates to obtain the final detection results, reporting an accuracy of 78.70%. An extended variation of PointRCNN is the part-aware and aggregation neural network (Part- $A^{2}$ Net). The part-aware stage, for the first time, fully utilizes free-of-charge part supervisions derived from 3D ground-truth boxes to simultaneously predict high-quality 3D proposals and accurate intra-object part locations. Then, the part-aggregation stage learns to re-score the box and refines the box location by exploring the spatial relationship of the pooled intra-object part locations. The reported accuracy reaches 79.40%. Yang et al. (2020b) introduced the 3D single-stage detection (3DSSD) framework, which employed a unique fusion sampling strategy that included farthest point sampling in both feature and Euclidean spaces. PointGNN (Shi and Rajkumar, 2020) extended the application of graph neural networks to 3D object detection. PV-RCNN (Shi et al., 2020) and its subsequent research (Shi et al., 2023) derived point-wise features from voxel abstraction networks to refine proposals generated by the 3D voxel backbone. Additionally, HVPR (Noh et al., 2021), a single-stage 3D detector, implemented an efficient memory module to enhance point-based features, achieving a better compromise between accuracy and efficiency. Qian et al. (2022) developed a lightweight region aggregation refine network (BANet) through local neighborhood graph construction, which resulted in more precise box boundary predictions.

#### 4.1.2 Cooperative object detection and fusion

Object detection from a single point of view of a single agent is definitely vulnerable to a series of sensor limitations that can significantly affect the outcome. These limitations entail occlusion, limited field-of-view, and low-point density at distant regions. The transition from isolated CPSs to CPSoSs, enabling the collaboration among various agents, offers the opportunity to tackle such problems. Chen et al. (2019b) proposed a cooperative sensing scheme where each CPS combines its sensing data with those of other connected vehicles to help enhance perception. Furthermore, to tackle the increased amount of data, the authors propose a sparse point-cloud object detection method. It is important to highlight that the agents share on-board V2V information and fuse these data locally. Feature-level fusion is examined in a follow-up work by Chen et al. (2019a). The authors propose F-Cooper framework, a method that improves the autonomous vehicle’s detection precision without introducing much computational overhead. This framework aims to utilize the capacity of feature maps, especially for 3D LiDAR data generated by autonomous vehicles as the feature maps are used for object detection only by single vehicles. F-Cooper is an end-to-end 3D object detection system with feature-level fusion supporting voxel feature fusion and spatial feature fusion. Voxel feature fusion achieves almost the same detection precision improvement compared to the raw-data level fusion solution, which offers the ability to dynamically adjust the size of feature maps to be transmitted. A unique characteristic of F-Cooper is that it can be deployed and executed on in-vehicle and roadside edge systems. Hurl et al. (2020) proposed TruPercept to tackle malicious attacks against cooperative perception systems. In their trust scheme, each agent reevaluates the detections originating from its neighboring agents using data from its position and perspective. Arnold et al. (2020) proposed a central system that fuses data from multiple infrastructure sensors, facilitating the management of both sensor and processing costs through shared resources while addressing evaluations of pose sensor configurations, the number of sensors, and the sensor field-of-view. The authors deploy VoxelNet (Zhou and Tuzel, 2018) and claim to have reached an average precision score of $AP_{3D}=98\%$ for the early fusion strategy and $AP_{3D}=81\%$ for the late fusion strategy in a T-Junction. In a more recent study, Guo et al. (2021) proposed cooperative spatial feature fusion (CoFF) to address F-Cooper limitations. F-Cooper’s Chen et al. (2019a) *maxout* function treats feature maps from different sources and conditions similarly, leading to misclassifications. CoFF calculates and assigns importance weights to the received feature maps based on the data available to the ego vehicle. The authors claim to reach a 90% improvement in average precision for far object cases with respect to F-Cooper.

### 4.2 Cooperative localization, cooperative path planning, and SLAM

Unmanned vehicles, either ground (UGV), aerial (UAV), or underwater (UUV), are prominent CPSoSs. Typical examples include autonomous vehicles and robots, operating for a variety of different civilian and military challenging tasks. At the same time, the prototyping of 5G and V2X (e.g., V2V and V2I) related communication protocols enables the close collaboration of vehicles to address their main individual or collective goals. Autonomous vehicles with inter-communication and network abilities are known as connected and automated vehicles (CAVs), being part of the more general concept of connected CPSoSs. The main focus of CAV’s related technologies is to increase and improve the safety, security, and energy consumption of (cooperative or not) autonomous driving by the strict control of the vehicle’s position and motion (Montanaro et al., 2018). At a higher level, CAV have the potential for a further enhancement of the transportation sector’s overall performance.

Perception and scene analysis ability are fundamental for a vehicle’s reliable operation. Computer vision-based object detection and tracking should be seen as a first (though necessary) pre-processing step, feeding more sophisticated operational modules of vehicles (Eskandarian et al., 2021). The latter is imperative to have accurate knowledge of both its own and its neighbors’ (vehicles, pedestrians, or static landmarks) position in order to design efficiently the future motion actions, i.e., to determine the best possible velocity, acceleration, yaw rate, etc. These motion actions primarily focus on, e.g., keeping safe inter-vehicular distances, eco-friendly driving by reducing gas emissions, etc. The above challenges can be addressed in the context of localization, SLAM, and Path planning, which are discussed below.

#### 4.2.1 Cooperative localization

The localization module is responsible for providing absolute position information to the vehicles. Global Navigation Satellite Systems (GNSSs), like GPS, Beidou, Glonass, etc., are usually exploited for that purpose. The GPS sensor is currently employed as the most common commercial device. It is straightforward to couple or fuse GPS information with IMU readings (Noureldin et al., 2013) to design a complete inertial navigation system (INS) providing positioning, velocity, and timing (PVT) solutions. The IMU sensor consists of gyroscopes and accelerometers for measuring the yaw rate and acceleration (in x,y,z directions) of vehicles. Additionally, odometers and wheel sensors (Skog and Handel, 2009) can also be utilized. However, even highly reliable IMU sensors suffer from accumulative or drift error, significantly reducing their consistency as the vehicle is moving. Another limitation of stand-alone GPS localization is directly related to GPS itself. Its accuracy is highly degraded in dense urban canyons or tunnels (Kuutti et al., 2018), even exceeding 10 m errors. The main sources of GPS signal degradation are due to Noureldin et al. (2013) satellite clock error, receiver clock error, ionosphere delay, tropospheric delay, multi-path, etc. Moreover, it is vulnerable to cyber-attacks (Ren et al., 2020), like spoofing or jamming. The former causes an intentionally “wrong” position, even kilometers away from the expected GPS measurement. The latter poses a rather more severe threat since it totally blocks the GPS signal. Several alternative approaches relying on ground base stations have been developed for enhancing localization accuracies, such as assisted GPS (AGPS) or differential GPS (DGPS). However, they are also susceptible to multi-path effects and signal blockage (Alam and Dempster, 2013). The desired localization error, as it has been reported in the literature, should be lower than 1 m (where in-lane accuracy) (Neto et al., 2020) to meet the standards of autonomous driving. For example, if a vehicle is localized on the curb instead of the road, it may lead to a serious accident with pedestrians or other vehicles. Therefore, it is quite clear that for obtaining the desired positioning solutions, other types of advanced sensors, like LiDAR, cameras, and RADAR, must be additionally taken into account. Moreover, the emergence of V2V communications in the context of the Internet of Things (IoT) facilitates the exploitation of both onboard and off-board information in order to design a more robust localization system. This collaborating multi-modal fusion of heterogeneous measurements is known as cooperative localization (CL), a rather recent and very promising technique that can tackle the limitations and drawbacks of GPS/IMU localization. Each vehicle can now receive external information (like absolute position, relative distance, velocity, and acceleration) from nearby vehicles, infrastructure, or pedestrians, effectively assisting its localization system.

There are many existing works (Kuutti et al., 2018; Buehrer et al., 2018; Wymeersch et al., 2009; Safavi et al., 2018; Gao, 2019) that survey-related aspects, challenges, and algorithms of CL. For example, Kuutti et al. (2018) provided an overview of current trends and future applications of localization (not only CL) in autonomous vehicle environments. The discussed techniques are mainly distinguished on the basis of the utilized sensor. Ranging measurements like relative distance and angle can also be extracted through the V2V abilities of CL. Common ranging techniques include time of arrival (TOA), angle of arrival (AoA), time difference of arrival (TDOA), and received signal strength (RSS). The works of Buehrer et al. (2018), Wymeersch et al. (2009) delve into detailed mathematical modeling of CL tasks. More specifically, Buehrer et al. (2018) exploit various criteria to categorize related algorithms:

• **Measurement type:** The sensor or ranging technique being used for localization. V2V communications enable different ranging methods to be used (as mentioned above).

• **Centralized vs. distributed:** Centralized algorithms require nodes/vehicles of the network to broadcast their measurements to a fusion center (e.g., cloud or some leader-vehicle), responsible for all the computations. Although higher accuracy can be achieved, limitations like communication overhead, computational power, network size, and fusion center malfunctioning must be taken into account. On the contrary, with distributed processing architecture, the computations are assigned to each vehicle, which interacts only with close neighbors.

• **One-shot vs. tracking:** One-shot refers to methods that do not exploit any past information. On the other hand, tracking has to do with algorithms that, apart from measurements, employ kinematic models in order to approximate the actual movement of vehicles. Tracking methods exploit Bayesian estimators, as mentioned below.

• **Fusion estimator:** Multi-modal fusion is vital for increased location estimation accuracy. Fusion can be effectively performed using well-known estimators like least squares (LS), maximum likelihood (ML), minimum mean square error (MMSE), and maximum a posteriori (MAP). The one-shot ML estimator coincides with (weighted by measurement noise variance) LS when the measurements are corrupted by Gaussian noise. MMSE and MAP are common Bayesian estimators that treat the unknown vehicle’s position as a random variable instead of a deterministic value as one-shot do. Kalman, extended Kalman, and unscented Kalman Filters (KF, EKF, and UKF) are prominent examples of MMSE estimators. Belief propagation and factor graph optimization are also important MAP tools.

**FIGURE 2:**
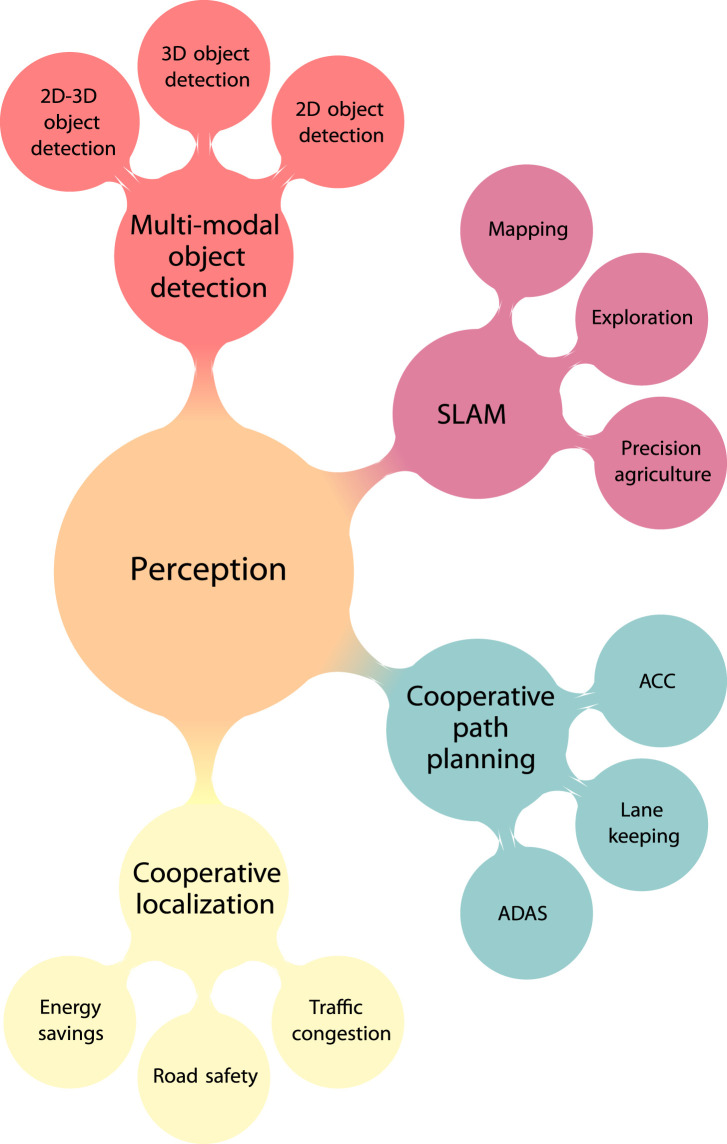
Key components of the perception layer in CPSoSs.

Wymeersch et al. (2009) formulated a distributed gradient descent (GD) algorithm as an LS solution and the Bayesian factor graph approach of the sum–product algorithm over wireless networks (SPAWNs). In general, distributed and tracking/Bayesian algorithms are more attractive to perform CL. An overview of distributed localization algorithms in IoT is also given by Safavi et al. (2018). Additionally, the authors discuss the proposed distributed geometric framework of DILOC, as well as the extended versions of DLRE and DILAND, which facilitate the design of a linear localization algorithm. These methods require the vehicle to be inside the convex full formed by three neighboring anchors (nodes with known and fully accurate positions) and to compute its barycentric coordinates with respect to neighbors. However, major challenges are related to mobile scenarios due to varying topologies, as well as how feasible the presence of anchors will be in automotive applications. An interesting approach is discussed by Meyer et al. (2016), where mobile agents, in general, try to cooperatively estimate their position and track non-cooperative objects. The authors developed a distributed particle filter-based belief propagation approach with message passing although they consider the presence of anchor nodes. Furthermore, the computational and communication overhead may be a serious limitation toward real-time implementation. Soatti et al. (2018) proposed a novel distributed technique to improve the stand-alone GNSS accuracy of vehicles. Once again, non-cooperative objects or features (e.g., trees and pedestrians) are exploited in order to improve location accuracy. Features are cooperatively detected by vehicles using their onboard sensors (e.g., LiDAR), where a perfect association is assumed. These vehicle-to-feature measurements are fused with GNSS in the context of a Bayesian message-passing approach and KF. Experimental evaluation was assessed using the SUMO simulator; however, the number of detected features, as well as communication overhead, should be taken into serious account. The work of Brambilla et al. (2020) extends that of Soatti et al. (2018) by proposing a distributed data association framework for features and vehicles. Data association was based on belief propagation. Validation was performed in realistic urban traffic conditions. The main aspect of Brambilla et al. (2020) and Soatti et al. (2018) is that vehicles must reach a consensus about feature states in order to improve their location. Graph Laplacian CL has been introduced in Piperigkos et al. (2020b) and Piperigkos et al. (2020a). Centralized or distributed Laplacian localization formulates an LS optimization problem, which fuses the heterogeneous inter-vehicular measurements along with the V2V connectivity topology through the linear Laplacian operator. EKF- and KF-based solutions have been proposed for addressing CL in tunnels (Elazab et al., 2017; Yang et al., 2020a) when the GPS signal may be blocked. A distributed robust cubature KF enhanced by Huber M-estimation is presented by Liu et al. (2017a). The method is used to tackle the challenges of data fusion in the presence of outliers. Pseudo-range measurements from satellites are also considered during the fusion process. Zhao et al. (2020) developed a distributed Bayesian CL method for localizing vehicles in the presence of non-line-of-sight range measurements and spoofed vehicles. They focused primarily on ego vehicle location estimation and abnormal vehicle detection rates. Potential applications of localization in various domains like wireless sensor networks (WSNs), intelligent transportation systems (ITS), and robotics are demonstrated in Figure 2. Table 1 summarizes the above mentioned works.

**TABLE 1:** Cooperative localization methods.

Fusion algorithm(s)	Survey	Centralized solution	Distributed solution	Benefits	Limitations	Reference
LS, GD, and SPAWN	—	—	✓	Two state-of-the art algorithms	Large number of iterations and information exchange are required to reach good solutions	Wymeersch et al. (2009)
Particle filter-based belief propagation	—	—	✓	Distributed tracking of mobile nodes and non-cooperative objects	Nodes have to reach a consensus on objects’ position	Meyer et al. (2016)
EKF	—	—	✓	Overall location estimation under harsh conditions and realistic network simulation	Lacks evaluation for the individual vehicle	Elazab et al. (2017)
Cubature KF and Huber M-estimation	—	—	✓	Robust location estimation in the presence of measurement outliers	Not considering the impact of dynamic VANET’s topology	Liu et al. (2017a)
—	✓	—	—	Complete survey of different fusion algorithms and technologies for CL	—	Buehrer et al. (2018)
—	✓	—	—	Complete survey of different fusion algorithms and technologies for CL, including SLAM methods	—	Kuutti et al. (2018)
Geometric algorithms	✓	—	✓	Linear and distributed approach based on sophisticated selection of neighbors	Developed mainly for static scenarios	Safavi et al. (2018)
Gaussian message passing and KF	—	✓	✓	Distributed CL method relying on the cooperative detection of features	Vehicles have to reach a consensus on features’ position	Soatti et al. (2018)
—	✓	—	✓	Detailed book about the current and potential status of CL methods	—	Gao (2019)
Particle filter-based belief propagation	—	—	✓	Distributed data association approach	Vehicles have to reach a consensus on features’ position	Brambilla et al. (2020)
Graph Laplacian processing	—	✓	—	Fusion of three measurement modalities via linear LS	No motion model is concerned	Piperigkos et al. (2020b)
Graph Laplacian processing	—	✓	✓	Fusion of three measurement modalities via linear LS	No motion model is concerned	Piperigkos et al. (2020a)
KF and ML	—	—	✓	Effective and simple implementation of cooperative awareness	Measurement model is rather abstract, not discussing in detail how it can be formulated	Yang et al. (2020a)
Bayesian approach	—	—	✓	Accurate location estimation under harsh conditions	Only ego vehicle location is assessed	Zhao et al. (2020)

#### 4.2.2 SLAM

Simultaneous localization and mapping (SLAM) is also a relevant task of localization. It refers to the problem of mapping an environment using measurements from sensors (e.g., a camera or LiDAR) onboard the vehicle or robot while at the same time estimating the position of that sensor relative to the map. Although when stated in this way, SLAM can appear to be quite an abstract problem robust, and efficient solutions to the SLAM problem are critical to enabling the next wave of intelligent mobile devices. SLAM, in its general, form tries to estimate over a time period the poses of the vehicle/sensor and the landmarks’ position of the map, given control input measurements provided by odometry sensors onboard the vehicle and measurements with respect to landmarks. Therefore, we have mainly two subsystems: the front-end, which detects the landmarks of the map and correlates them with the poses, and the back-end, which casts an optimization problem in order to estimate the pose and the location of landmarks. SLAM techniques can be distinguished to either visual or LiDAR based odometry (VO and LO) solutions, reflecting camera or LiDAR as the main sensor to be exploited:

• To compute the local position and motion of a camera, VO algorithms must estimate the transformation that the camera undergoes between the current frame and a reference frame. The reference frame can be defined by the previous frame in the input sequence, some key frame in the recent past, or a collection of frames from the recent past. In each case, the task is to estimate the transformation that takes information in the camera’s current frame into the frame of reference of the past frame(s). This task can be seen as an optimization problem where the cost is given by the residual between the information measured in the current frame and the corresponding information derived and reprojected from the reference frames. The vast majority of VO algorithms use feature-based approaches (Klein and Murray, 2007). The image is decomposed into a sparse set of interesting points, with each interest point’s location described by a feature vector that remains invariant to camera transformations. The feature vectors are associated with the input frame and reference to form a set of geometric constraints from which we can derive the camera motion and scene structure. In this case, the cost function is formulated by the difference between the measured reprojection locations of these interest points between frames, referred to as the reprojection error. However, some known limitations of feature-based methods include i) extraction of interesting points and feature vectors may be expensive (using well-known algorithms like SHIFT or SURF), ii) they are prone to errors in areas where there is a low number of interesting points, etc. On the contrary, dense or direct VO approaches (Steinbrücker et al., 2011; Whelan et al., 2013) focus on minimizing the (geometric) reprojection error, aiming to directly minimize the photometric error between pixels in the optimization problem. State-of-the-art VO algorithms include Direct Sparse Odometry (DSO) (Engel et al., 2018), ORB-SLAM (Mur-Artal et al., 2015), and ORB-SLAM2 (Mur-Artal and Tardos, 2017).

• The LiDAR sensor provides dense 3D point clouds of the vehicle’s surroundings. The goal of LO is to estimate the pose of the vehicle by accumulating the transformation between consecutive frames of 3D point clouds. The existing LO solutions can be divided into two groups: point-wise and feature-wise methods. Point-wise methods estimate the relative transformation directly using the raw 3D points, while feature-wise methods try to utilize more sophisticated characteristics of the point cloud such as the edge and planar feature points. The most well-known pointwise LO method is the iterative close point (ICP) (Besl and McKay, 1992). ICP operates at a point-wise level and directly matches two frames of the point cloud by finding the correspondences. One of the major drawbacks of the ICP is that when the frames include large quantities of points, ICP may suffer from a high computational load arising from the point cloud registration. Many variants of ICP have been proposed to improve its efficiency and accuracy, such as the trimmed ICP (Makihara et al., 2002) and normal ICP (Serafin and Grisetti, 2015). To avoid the high computational load resulting from using the entire set of raw points, the feature-based LO methods extract a set of representative features from the raw points. The fast point feature histogram (FPFH) was proposed by Rusu et al. (2009) to extract and describe important features. The FPFH enables the exploration of the local geometry and the transformation is optimized by matching the one-by-one FPFH-based correspondence. Another well-known feature-based LO method is LOAM (Zhang and Singh, 2014). Theoretically, LOAM integrates the properties of both the point- and feature-wise methods. On the one hand, to decrease the computational load of typical ICP, LOAM proposed to extract two types of feature points, the edge and planar, respectively. The extraction of the feature is simply based on the smoothness of a small region near a given feature point. Different from the FPFH which provides multiple categories of features based on its descriptors, LOAM involves only two feature groups. Another popular variant of LOAM is Lego-LOAM (Shan and Englot, 2018).

Cooperative SLAM approaches are, in general, more immature with respect to CL since they are usually applied in indoor experimental environments with small-scale robots. A thorough overview of multiple-robot SLAM methods is provided by Saeedi et al. (2016), focusing mainly on agents equipped with cameras or 2D LiDARs (Mourikis and Roumeliotis, 2006; Zhou and Roumeliotis, 2008; Estrada et al., 2005). In addition, cooperative SLAM approaches using 3D LiDAR sensors are discussed by Kurazume et al. (2017), Michael et al. (2012), and Nagatani et al. (2011). Table 2 summarizes the above mentioned SLAM-based methods.

**TABLE 2:** SLAM methods based on VO and LO solutions.

Camera	LiDAR	Benefits	Limitations	Reference
	✓	Fundamental work	High computational load	ICP (Besl and McKay, 1992)
—	✓	Variant of ICP	Improves the computational complexity of ICP	TICP (Makihara et al., 2002)
✓	—	Fundamental feature-based approach	Challenging the extraction of feature points	Klein and Murray (2007)
—	✓	Exploits a set of representative features from the raw point cloud	Lacks evaluation under different weather and lighting conditions	FPFH (Rusu et al., 2009)
✓	—	Directly minimize the photometric error between pixels	Sensitive to image noise	Steinbrücker et al. (2011)
✓	—	Directly minimize the photometric error between pixels	Sensitive to image noise	Whelan et al. (2013)
—	✓	State-of-the-art LO solution	Lacks evaluation under different weather and lighting conditions	LOAM (Zhang and Singh, 2014)
✓	—	State-of-the-art VO solution	Lacks evaluation under different weather and lighting conditions	ORB-SLAM (Mur-Artal et al., 2015)
—	✓	Variant of ICP	Improves the computational complexity of ICP	NICP (Serafin and Grisetti, 2015)
✓	—	State-of-the-art VO solution	Lacks evaluation under different weather and lighting conditions	ORB-SLAM2 (Mur-Artal and Tardos, 2017)
✓	—	State-of-the-art VO solution	Lacks evaluation under different weather and lighting conditions	DSO (Engel et al., 2018)
—	✓	State-of-the-art LO solution	Lacks evaluation under different weather and lighting conditions	LeGO-LOAM (Shan and Englot, 2018)

#### 4.2.3 Cooperative path planning

Connected advanced driver assistance systems (ADASs) help reduce road fatalities and have received considerable attention in research and industrial societies (Uhlemann, 2016). Recently, there has been a shift of focus from individual drive-assist technologies like power steering, anti-lock braking systems (ABS), electronic stability control (ESC), and adaptive cruise control (ACC) to features with a higher level of autonomy like collision avoidance, crash mitigation, autonomous drive, and platooning. More importantly, grouping vehicles into platoons (Halder et al., 2020; Wang et al., 2019a) has received considerable interest since it seems to be a promising strategy for efficient traffic management and road transportation, offering several benefits in highway and urban driving scenarios related to road safety, highway utility, and fuel economy.

To maintain the cooperative motion of vehicles in a platoon, the vehicles exchange their information with the neighbors using V2V and V2I (Hobert et al., 2015). The advances in V2X communication technology (Hobert et al., 2015; Wang et al., 2019b) enable multiple automated vehicles to communicate with one another, exchanging sensor data, vehicle control parameters, and visually detected objects facilitating the so-called 4D cooperative awareness (e.g., identification/detection of occluded pedestrians, cyclists, or vehicles).

Several works have been proposed for tackling the problems of cooperative path planning. Many of them focus on providing spacing policy schemes using both centralized and decentralized model predictive controllers. However, very few take into account the effect of network delays, which are inevitable and can significantly deteriorate the performance of distributed controllers.

Viana et al. (2019) presented a unified approach to cooperative path-planning using nonlinear model predictive control with soft constraints at the planning layer. The framework additionally accounts for the planned trajectories of other cooperating vehicles, ensuring collision avoidance requirements. Similarly, a multi-vehicle cooperative control system is proposed by Bai et al. (2023) and Kuriki and Namerikawa (2015) with a decentralized control structure, allowing each automated vehicle to conduct path planning and motion control separately. Halder et al. (2020) presented a robust decentralized state-feedback controller in the discrete-time domain for vehicle platoons, considering identical vehicle dynamics with undirected topologies. An extensive study of their performance under random packet drop scenarios is also provided, highlighting their robustness in such conditions. Viana and Aouf (2018) extended decentralized MPC schemes to incorporate the predicted trajectories of human-driving vehicles. Such solutions are expected to enable the co-existence of vehicles supporting various levels of autonomy, ranging from L0 (manual operation) to L5 (fully autonomous operation) (Taeihagh and Lim, 2019). Furthermore, a distributed motion planning approach based on the artificial potential field is proposed by Xie et al. (2022b), where its innovation is related to developing an effective mechanism for safe autonomous overtaking when the platoon consists of autonomous and human-operated vehicles.

In additional to the cooperative path planning mechanisms, spacing policies and controllers have also received increased interest in ensuring collision avoidance by regulating the speeds of the vehicles forming a platoon. Two different types of spacing policies can be found in the literature, i.e., the constant-spacing policy (Liu et al., 2017; Shen et al., 2022) and the constant-time-headway spacing policy (e.g., focusing on maintaining a time gap between vehicles in a platoon resulting in spaces that increase with velocity) (Wang et al., 2023a). In both categories, most works use a one-direction control strategy. At this point, it should be mentioned that in a one-directional strategy, the vehicle controller processes the measurements that are received from leading vehicles. Similarly, a bidirectional platoon control scheme takes into consideration the state of vehicles in front and behind (see Ghasemi et al., 2013). In most of the cooperative platooning approaches, the vehicle platoons are formulated as double-integrator systems that deploy decentralized bidirectional control strategies similar to mass–spring–damper systems. This model is widely deployed since it is capable of characterizing the interaction of the vehicles with uncertain environments and, thus, is more efficient in stabilizing the vehicle platoon system in the presence of modeling errors and measurement noise. However, it should be noted that the effect of network delays on the performance of such systems has not been extensively studied. Time delays, including sensor detective delay, braking delay, and fuel delay, not only seem to be inevitable but are also expected to deteriorate significantly the performance of the distributed controllers. Table 3 summarizes the above mentioned Path planning-based methods.

**TABLE 3:** Path planning methods.

Cooperativepathplanning	Spacing controllermechanism	Year	Reference
—	✓	2013	Ghasemi et al. (2013)
✓	—	2015	Kuriki and Namerikawa (2015)
—	✓	2017	Liu et al. (2017)
✓	—	2018	Viana and Aouf (2018)
✓	—	2019	Viana et al. (2019)
✓	—	2019	Taeihagh and Lim (2019)
✓	—	2022	Xie et al. (2022b)
—	✓	2022	Shen et al. (2022)
✓	—	2023	Bai et al. (2023)
—	✓	2023	Wang et al. (2023a)

### 4.3 Human centric perception

**FIGURE 3:**
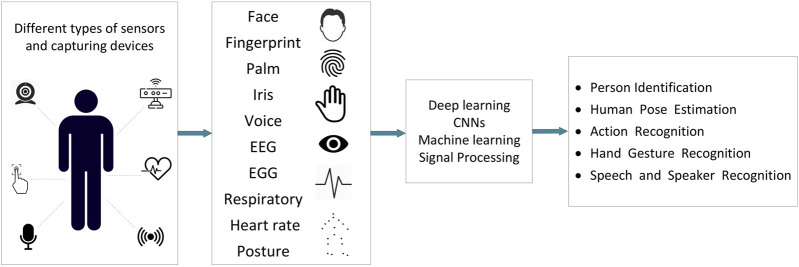
Human biometrics and applications in CPSoSs.

Humans, as a part of a CPSoS, play an important role in the functionality of the system. The humans’ role in such complicated systems (e.g., CPSoSs) is vital since they react and collaborate with the machines, providing them with useful feedback and affecting the way that these systems work. Humans can provide valuable input both in an active (on purpose) or a passive (without consideration) way (Figure 3). For example, an input such as a gesture or voice can be used as an order or command to control the operation of a system via an HMI. On the other hand, pose estimation or biometrics, like heart rate, could be taken into account by a decision component, resulting in a corresponding change in the system’s functionality for security reasons (e.g., when a user’s fatigue has been detected).

The following sections present some human-related inputs (e.g., behavior and characteristics) that can be beneficially used in CPSoSs according to the literature.

• **Biometrics and biometric recognition.** The most well-known and most frequently used biometrics related to humans are face, fingerprint, iris, EEG, EGG, respiratory, and heart rate. Some of them are unique for each person, so they can be used for human identification, while others can be used for monitoring the humans’ state or the special cognitive situation of a specific time period. The use of biometrics covers a large variety of tasks and applications in CPSoSs.

Regarding the face of a human as a biometric, the related tasks can be face detection (Claudi et al., 2013; Isern et al., 2020; Chen et al., 2016; Galbally et al., 2019), face alignment (Kaburlasos et al., 2020a; Kaburlasos et al., 2020b), face recognition (Makovetskii et al., 2020), face tracking (Li et al., 2016b; Pereira Passarinho et al., 2015), face classification/verification (Arvanitis et al., 2016), and face landmarks extraction (Jeong et al., 2018; Eskimez et al., 2020; Lee et al., 2020). Fingerprint (Okpara and Bekaroo, 2017; Valdes-Ramirez et al., 2019; Preciozzi et al., 2020), palmprint (Wang et al., 2006), and iris/gaze (Vicente et al., 2015; Lai et al., 2015) are mainly used for user’s identification tasks due to their uniqueness for each person. EEG (Laghari et al., 2018; Pandey et al., 2020; Lou et al., 2017), EGG, respiratory (Jiang et al., 2020; Saatci and Saatci, 2021; Meng et al., 2020), and heart rate (Rao et al., 2012; Prado et al., 2018; Majumder et al., 2019) are used for the user’s state monitoring. In addition to the fact that they can provide valuable information, their usage in real applications is difficult to apply due to the special wearable devices that it is required for the capturing.

The choice of which specific biometric to utilize depends on the use case scenario, including factors such as the availability and feasibility of using a sensor (e.g., whether it will be placed in a stationary location or worn constantly during the operation), the special power consumption needs of each sensor, the accuracy, and the latency. One other important issue that needs to be taken under serious consideration before the use of a biometric in real systems is the privacy and security of these sensitive data since they must be protected via encoding in order to be anonymously stored or used.

• **Person identification.** Person identification is a common image retrieval problem, where the objective is the recognition of a person’s identity using only a single image captured by a camera.

Generally, the person identification task is a more complicated and challenging problem in comparison to the face identification since face identification is applied in a more controlled environment (e.g., use of a smaller captured frame, the user has to remove glasses, hats, and other accessories to be identified). On the other hand, person identification has to deal with more complex issues like the different points of view, light and weather conditions, different resolutions of the camera, types of clothes, and a large variety of background contexts. Person identification has shown great usability in applications related to CPSoSs, mostly for security purposes. Its utility has been marked specifically when it is applied “in the wild” and in uncontrolled environments where other biometrics are not feasible to be used due to technical constraints. Nowadays, approaches usually use deep networks to perform reliable and accurate results.

Li et al. (2020a) proposed an additive distance constraint approach with similar label loss to learn highly discriminative features for person re-identification. Ye and Yuen (2020) proposed a deep model (PurifyNet) to address the issue of the person re-identification task with label noise, which has limited annotated samples for each identity. Li et al. (2020b) used an unsupervised re-identification deep learning approach capable of incrementally discovering discriminative information from automatically generated person tracklet data. Table 4 and Table 5 summarize relevant datasets for the face recognition, detection, and facial landmark extraction problems.

**TABLE 4:** Datasets for face recognition, detection, and facial landmark extraction tasks.

Dataset	Short description	Link of the dataset	Paper name
Helen	Helen dataset consists of 2,330 images (400 × 400 pixels) with labeled facial components, which are manually annotated, containing contours near the eyes, eyebrows, nose, lips, and jawline	http://www.ifp.illinois.edu/∼vuongle2/helen/	Interactive Facial Feature Localization (Le et al., 2012)
AFW	AFW (Annotated Faces in the Wild) is a face detection dataset consisting of 205 images with 468 faces. Each face image is labeled with at most 6 landmarks with visibility labels, as well as a bounding box	https://www.ics.uci.edu/∼xzhu/face/	Face detection, pose estimation, and landmark localization in the wild (Zhu and Ramanan, 2012)
300W	The 300-W dataset consists of 300 indoor and 300 outdoor “in the wild” images, covering a large variety of identities, expressions, illumination conditions, poses, occlusion, and face sizes	https://ibug.doc.ic.ac.uk/resources/300-W/	300 Faces in-the-Wild Challenge: The First Facial Landmark Localization Challenge (Sagonas et al., 2013)
LFPW	The Labeled Face Parts in the Wild (LFPW) consists of 1,432 faces from images which are downloaded from the web (e.g., google.com, flickr.com, and yahoo.com)	https://neerajkumar.org/databases/lfpw/	Localizing parts of faces using a consensus of exemplars (Belhumeur et al., 2011)
AFLW	The Annotated Facial Landmarks in the Wild (AFLW) consists of 25,000 faces that are annotated with up to 21 landmarks per image. The images have been gathered from Flickr, covering a large variety of poses, expressions, ethnicities, ages, genders, and environmental conditions	https://www.tugraz.at/institute/icg/research/team-bischof/lrs/downloads/aflw/	Annotated Facial Landmarks in the Wild: A large-scale, real-world database for facial landmark localization (Köstinger et al., 2011)
AFLW 2000-3D	AFLW 2000-3D dataset consists of 2,000 images that have been annotated using 68 points representing 3D facial landmarks. This dataset is usually used for the evaluation of 3D facial landmark detection models	http://www.cbsr.ia.ac.cn/users/xiangyuzhu/projects/3DDFA/main.htm	Face Alignment Across Large Poses: A 3D Solution (Zhu et al., 2016)
300-VW	300 Videos in the Wild (300-VW) is a dataset for evaluating facial landmark tracking algorithms in the wild. Each video of this dataset is almost 1 min in duration (at 25–30 fps). Each frame of all videos has been annotated in the same way as the 300-W dataset	https://ibug.doc.ic.ac.uk/resources/300-VW/	Offline Deformable Face Tracking in Arbitrary Videos (Chrysos et al., 2015)
COCO-WholeBody	This dataset is an extension of COCO dataset, covering a whole-body annotation (i.e., face, hand, and feet)	https://github.com/jin-s13/COCO-WholeBody	Whole-Body Human Pose Estimation in the Wild (Jin et al., 2020)
MALF	MALF consists of 5,250 images with 11,931 faces in total. This dataset is the first face detection dataset that supports fine-gained evaluation	http://www.cbsr.ia.ac.cn/faceevaluation/	Fine-grained Evaluation on Face Detection in the Wild (Yang et al., 2015)
FDDB	FDDB dataset consists of 2,845 images with 5,171 annotated faces	http://vis-www.cs.umass.edu/fddb/index.html	FDDB: A Benchmark for Face Detection in Unconstrained Settings (Jain and Learned-Miller, 2010)

**TABLE 5:** Datasets of images with the iris.

Dataset	Short description	Link of the dataset	Paper name
UBIRIS.v2	The UBIRIS.v2 dataset consists of 11,102 images of the iris that were captured from 261 subjects, with 10 images for each subject. The images were acquired using a variety of different conditions like distance, motion, and different visible wavelengths. They have also been affected by real noise	http://iris.di.ubi.pt/ubiris2.html	The UBIRIS.v2: A Database of Visible Wavelength Iris Images Captured On-the-Move and At-a-Distance (Proenca et al., 2010)
OpenEDS	Open Eye Dataset (OpenEDS) consists of images with eyes captured using a virtual-reality head display. This dataset was collected from 152 individual participants and is divided into four subsets	https://research.fb.com/programs/	OpenEDS: Open Eye Dataset (Garbin et al., 2019)

• **Human pose estimation and action recognition.**

Human pose estimation and action recognition have been proved to be particularly valuable tasks in modern video-captured applications related to CPSoSs. They can be utilized in a variety of fields such as ergonomics assessment, safe training of new operators, fatigue and drowsiness detection of the user, HMIs, and the prediction of an operator’s next action for avoiding accidents by changing the operation of a machine. They are also useful for monitoring dangerous movements in insecure workspace areas.

A restriction that can negatively affect and obstruct the quality of the results of these tasks is the limited coverage area of the camera. Nevertheless, this limitation can be overcome using new types of sensors and tools like IMUs and whole-body tracking systems (e.g., SmartsuitPro and Xsens) (Hu et al., 2017).

Islam et al. (2019) presented an approach that exploits visual cues from human pose to solve industrial scenarios for safety applications in CPSs. El-Ghaish et al. (2018) integrated three modalities (i.e., 3D skeletons, body part images, and motion history images) into a hybrid deep learning architecture for human action recognition. Nikolov et al. (2018) proposed a skeleton-based approach utilizing spatio-temporal information and CNNs for the classification of human activities. Deniz et al. (2020) presented an indoor monitoring reconfigurable CPS that uses embedded local nodes (Nvidia Jetson TX2), proposing learning architectures to address human action recognition. Table 6 and Table 7 summarize relevant datasets for the pose estimation and action recognition problem.

**TABLE 6:** Datasets for pose estimation.

Dataset	Short description	Link of the dataset	Paper name
COCO	The Microsoft Common Objects in Context (MS COCO) consists of 328,000 images. This dataset is a general-proposed, large-scale object detection, segmentation, key-point detection, and captioning dataset containing labeled human’s poses	https://cocodataset.org/	Microsoft COCO: Common Objects in Context (Lin et al., 2014)
MPII	The MPII Human Pose Dataset consist of 25,000 images, of which 15,000 images are training samples, 3,000 images are validation samples, and the remaining 7,000 images are testing samples. The single-person poses are manually annotated with up to 16 body joints. The images are taken from YouTube videos, covering 410 different human activities	http://human-pose.mpi-inf.mpg.de/	2D Human Pose Estimation: New Benchmark and State of the Art Analysis (Andriluka et al., 2014)
DensePose	DensePose-COCO is a large-scale ground-truth dataset with image-to-surface correspondences, which are manually annotated from 50,000 images of the COCO dataset and train DensePose-RCNN, to densely regress part-specific UV coordinates within every human region at multiple frames per second	http://densepose.org/	DensePose: Dense Human Pose Estimation in the Wild (Güler et al., 2018)
LSP	The Leeds Sports Pose (LSP) dataset consists of 2,000 images of sportspersons in total gathered from Flickr, 1,000 for training and 1,000 for testing. This dataset is used for human pose estimation, and each image is annotated with 14 joint locations	https://dbcollection.readthedocs.io/en/latest/datasets/leeds_sports_pose_extended.html	Clustered Pose and Nonlinear Appearance Models for Human Pose Estimation (Johnson and Everingham, 2010)
JHMDB	JHMDB is a recognition dataset that consists of 960 video sequences belonging to 21 actions. This dataset is a subset of the larger HMDB51 dataset, which has been collected from digitized movies and YouTube videos	http://jhmdb.is.tue.mpg.de/	Towards Understanding Action Recognition (Jhuang et al., 2013)
Unite the People	Unite The People dataset is mainly used for 3D body estimation. The images come from an extended version of the LSP dataset, as well as the single person-tagged people from the MPII Human Pose Dataset. The images are labeled with different types of annotations such as segmentation labels, poses, or 3D representation	https://files.is.tuebingen.mpg.de/classner/up/	Unite the People: Closing the Loop Between 3D and 2D Human Representations (Lassner et al., 2017)

**TABLE 7:** Datasets of action recognition.

Dataset	Short description	Link of the dataset	Paper name
UCF101	This dataset consists of 13,320 video clips ( ∼ 27 h) from Youtube, classified into 101 categories and into 5 types (i.e., body motion, human–human interactions, human–object interactions, playing musical instruments, and sports)	https://www.crcv.ucf.edu/data/UCF101.php	UCF101: A Dataset of 101 Human Actions Classes From Videos in The Wild (Soomro et al., 2012)
Kinetics	It is a high-quality dataset of videos used for human action recognition. The dataset consists of approximately 500,000 labeled video clips of 10 s, covering 600 human action classes with at least 600 video clips for each action class	https://deepmind.com/research/open-source/kinetics	The Kinetics Human Action Video Dataset (Kay et al., 2017)
HMDB51	The HMDB51 is a dataset consisting of 6,849 video clips from 51 action categories (such as “jump,” “kiss,” and “laugh”). Each category containing at least 101 clips	https://serre-lab.clps.brown.edu/resource/hmdb-a-large-human-motion-database/	HMDB: A large video database for human motion recognition (Kuehne et al., 2011)
ActivityNet	The ActivityNet contains 200 different types of activities and a total of 849 h of videos collected from YouTube. It is one of the largest datasets based on the number of activity categories and videos	http://activity-net.org/	ActivityNet: A Large-Scale Video Benchmark for Human Activity Understanding (Heilbron et al., 2015)
NTU RGB + D	NTU RGB + D consists of 56,880 video clips of 60 action classes collected from 40 subjects. The actions can be generally divided into 3 categories: 40 daily actions (e.g., drinking, eating, and reading), 9 health-related actions (e.g., sneezing, staggering, and falling down), and 11 mutual actions (e.g., punching, kicking, and hugging)	http://rose1.ntu.edu.sg/datasets/actionrecognition.asp	NTU RGB + D: A Large Scale Dataset for 3D Human Activity Analysis (Shahroudy et al., 2016)
KTH	The KTH dataset contains six actions: walk, jog, run, box, hand-wave, and hand clap by 25 different individuals in different environments: outdoor (s1), outdoor with scale variation (s2), outdoor with different clothes (s3), and indoor (s4)	https://www.csc.kth.se/cvap/actions/	Recognizing Human Actions: A Local SVM Approach (Schuldt et al., 2004)
Composable activity dataset	This dataset consists of 693 annotated videos of activities in 16 classes performed by 14 individuals	https://ialillo.sitios.ing.uc.cl/ActionsCVPR2014/	Discriminative Hierarchical Modeling of Spatio-Temporally Composable Human Activities (Lillo et al., 2014)
HACS	HACS dataset contains 504 K videos (shorter than 4 min) collected from YouTube, categorized in 200 action classes. It is used in human action recognition	http://hacs.csail.mit.edu/	HACS: Human Action Clips and Segments Dataset for Recognition and Temporal Localization (Zhao et al., 2019a)

• **Hand gesture recognition.**

Hand gesture recognition tasks can be a very useful tool for interactions with machines or subsystems in CPSoSs (Horváth and Erdős, 2017), particularly in applications where the user is not allowed to have physical hand contact with a machine due to security reasons. This task mainly consists of three sequential steps, which are hand detection, hand tracking, and gesture recognition. Gesture recognition can occur either by a single image (i.e., static gesture recognition) or a sequence of images (i.e., dynamic gesture recognition). The first strategy looks more like a retrieval problem where the gesture of the image has to match with a known predefined gesture from a dataset of gestures. The second is a more complicated problem, but it is more useful since it can cover the requirements of a wider variety of real-life use cases (Choi and Kim, 2017). Gesture recognition is a very common task in human–computer interaction. Nonetheless, the recognition of complex patterns demands accurate sensors and sufficient computational power (Grützmacher et al., 2016). Additionally, we have to refer to the fact that visual computing plays an important role in CPSoSs, especially in these applications where the visual gesture recognition system relies on multi-sensor measurements (Posada et al., 2015; Aviles-Arriaga et al., 2006).

Horváth and Erdős (2017) presented a control interface for cyber–physical systems that interprets and executes commands in a human-robot shared workspace using a gesture recognition approach. Lou et al. (2016) tried to address the problem of personalized gesture recognition for cyber–physical environments, proposing an event-driven service-oriented framework. However, in other gesture recognition applications, a body-worn setup was proposed, which supplements the omnipresent 3 DoF motion sensors with a set of ultrasound transceivers (Putz et al., 2020). Table 8 summarizes relevant datasets for the hand and gesture recognition problems.

**TABLE 8:** Datasets for hand and gesture recognition.

Dataset	Short description	Link of the dataset	Paper name
HandNet	The HandNet dataset contains the depth images of 10 participants’ hands non-rigidly deforming in front of a RealSense RGB-D camera. The annotations were generated using a magnetic annotation technique. 6D pose is available for the center of the hand and the five fingertips (i.e., position and orientation of each)	http://www.cs.technion.ac.il/∼twerd/HandNet/	Rule of thumb: Deep derotation for improved fingertip detection (Wetzler et al., 2015)
EgoGesture	The EgoGesture dataset consists of 2,081 RGB-D videos, 24,161 gesture samples, and 2,953,224 frames from 50 distinct subjects	http://www.nlpr.ia.ac.cn/iva/yfzhang/datasets/egogesture.html	EgoGesture: A New Dataset and Benchmark for Egocentric Hand Gesture Recognition (Zhang et al., 2018)
NVGesture	The NVGesture dataset consists of 1,532 dynamic gestures categorized into 25 classes. The dataset is separated into 1,050 samples for training and 482 for testing. The application in which it can be used is for touchless driver controlling	https://ieeexplore.ieee.org/document/7780825	Online Detection and Classification of Dynamic Hand Gestures With Recurrent 3D Convolutional Neural Network (Molchanov et al., 2016)
IPN Hand	The IPN Hand is a dataset consisting of videos with sufficient size, variation, and real-world elements capable to be used by deep neural networks for training and evaluation. The application on which this dataset focuses is dynamic hand gesture recognition	https://github.com/GibranBenitez/IPN-hand	Real-time Hand Gesture Detection and Classification Using Convolutional Neural Networks (Köpüklü et al., 2019)
MLGEST-URE	MlGesture consists of more than 1,300 hand gesture videos from 24 participants and features 9 different hand gesture symbols. The dataset has been recorded in a car with five different sensor types at two different viewpoints, and it can be used for hand gesture recognition tasks	https://iiw.kuleuven.be/onderzoek/eavise/mlgesture/home	Low-latency hand gesture recognition with a low resolution thermal imager (Vandersteegen et al., 2020)

• **Speech and speaker recognition.**

Speech recognition is a sub-category of a more generic research area related to the domain of natural language processing (NLP). The main objective of speech recognition is to automatically translate the content of the entire speech (or the most significant part of it) into text or other recognizable forms from the computers. Assuming that the recording and processing of speech do not require a special sensor, but just a simple audio recorder, we can understand how easy to use this information is. Additionally, speech can be applied without any physical contact interaction, making it an ideal signal for HMI applications.

Speech recognition tasks can be utilized in the smart input system (Wang, 2020; Han et al., 2016), automatic transcription system (Chaloupka et al., 2012; Chaloupka et al., 2015), smart voice assistant (Subhash et al., 2020), computer-assisted speech (Tejedor-García et al., 2020), rehabilitation (Aishwarya et al., 2018; Mariya Celin et al., 2019), and language teaching.

Similar to the face recognition task that focuses on the recognition of an individual human using facial information, the speaker recognition task tries to achieve the same goal using the vocal tone information of the subject. Speaker recognition is one of the most basic components for human identification, which has various applications in many CPSoSs. Additionally, fusion schemes can be used combining both speaker recognition and face recognition for more secure integrations (Zhang and Tao, 2020).

A speaker recognition system consists of three separate parts, namely, the speech acquisition module, the feature extraction and selection module, and finally the pattern matching and classification module. In CPSoSs, the implementation of an automatic speech recognition system relies on a voice user interface so that humans can interact with robots or other CPS components. Nevertheless, this type of interface cannot replace the classical GUIs, but it can intensify them by providing, in some cases, a more efficient way of interaction.

Kozhirbayev et al. (2018) developed a technique to train a neural network (NN) on the extracted mel-frequency cepstral coefficient (MFCC) features from audio samples to increase the recognition accuracy of the short utterance speaker recognition system. Wang et al. (2020) tried to improve the robustness of speaker identification using a stacked sparse denoising auto-encoder. Table 9 summarizes relevant datasets for the speech recognition problem.

**TABLE 9:** Datasets for speech recognition.

Dataset	Short description	Link of the dataset	Paper name
LibriSpeech	This dataset consist of approximately 1,000 h of audiobooks	http://www.openslr.org/12	LibriSpeech: An ASR corpus based on public domain audio books (Panayotov et al., 2015)
Speech Commands	Speech Commands consists of 65,000 of 30 short words ∼ , one second long. It is a collection of spoken words by thousands of different people, designed for the training and evaluation of keyword spotting systems	https://ai.googleblog.com/2017/08/launching-speech-commands-dataset.html	Speech Commands: A Dataset for Limited-Vocabulary Speech Recognition (Warden, 2018)
MuST-C	MuST-C currently represents the largest publicly available multilingual corpus for speech translation from English into several languages. It covers eight languages. It consists of hundred hours of audio recordings from English TED Talks	https://ict.fbk.eu/must-c/	MuST-C: A multilingual corpus for end-to-end speech translation (Cattoni et al., 2021)
Common Voice	Common Voice is a dataset of 9,283 recorded hours that consists of audio files and corresponding text files including demographic metadata like age, sex, and accent	https://commonvoice.mozilla.org/en/datasets	Common Voice: A Massively-Multilingual Speech Corpus (Ardila et al., 2019)
Libri-Light	Libri-Light is a collection of over 60 K hours of spoken English suitable for training speech recognition systems under limited or no supervision	https://github.com/facebookresearch/libri-light	Libri-Light: A Benchmark for ASR with Limited or No Supervision (Kahn et al., 2020)
THCHS-30	THCHS-30 is a free Chinese speech database that can be used for speech recognition systems	http://166.111.134.19:7777/data/thchs30/README.html	THCHS-30: A Free Chinese Speech Corpus (Wang and Zhang, 2015)
VOICES	This dataset consists of speech recorded by far-field microphones in noisy room conditions for using in speech and signal processing approaches	https://registry.opendata.aws/lab41-sri-voices/	Voices Obscured in Complex Environmental Settings (VOICES) corpus (Richey et al., 2018)
LibriCSS	LibriCSS is a real recorded dataset that simulates conversations that are captured by far-field microphones	https://github.com/chenzhuo1011/libri_css	Continuous speech separation: dataset and analysis (Chen et al., 2020)
SPEECH-COCO	SPEECH-COCO contains 616,767 audios generated using text-to-speech (TTS) synthesis. The audio files are paired with images	https://zenodo.org/record/4282267	PEECH-COCO: 600k Visually Grounded Spoken Captions Aligned to MSCOCO Data Set (Havard et al., 2017)

## 5 Behavioral layer

In each CPSoS, human knowledge, senses, and expertise constitute important informative values that can be taken into account for the assurance of its operational excellence. However, a substantial concern that needs to be addressed at an early age of CPSoS evolution is determining how these abstract human features can be made accessible and understandable to the system.

A way to integrate the human as a separate component into a CPSoS is by introducing an anthropocentric mechanism, which is known in the literature as the HITL approach (Gaham et al., 2015; Hadorn et al., 2016). This mechanism allows a direct way for humans to continuously interact with the CPSoSs’ control loops in both directions of the system (i.e., taking and giving inputs).

Although common CPSoSs are human-centered systems (where human constitutes an essential part of the system), unfortunately, in many real cases, these systems still consider humans as external and unpredictable elements without taking their importance into deeper consideration. The central vision of the researchers and engineers is to create a human–machine symbiosis, integrating humans as holistic beings within CPSoSs. In this way, CPSoSs have to support a tight bond with the human element through HITL controls, taking into account human features like intentions, psychological and cognitive states, emotions, and actions, all of which can be deduced through sensor data and signal processing approaches.

**FIGURE 4:**
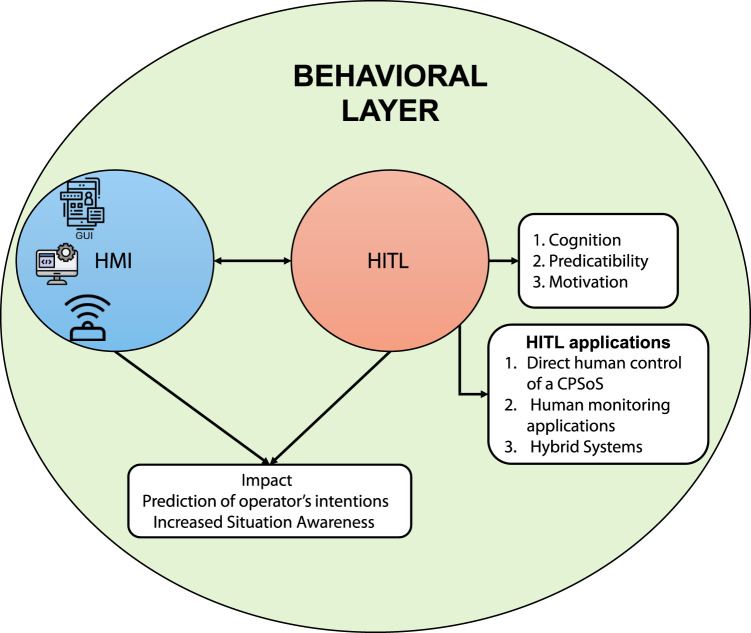
Key components of the behavioral layer in CPSoSs.

HITL systems integrate human feedback into the control loop of a system, allowing humans to interact with and influence automated processes in real-time. In self-driving vehicles, haptic teleoperation enables remote operators to control the vehicle with the sensation of touch. For instance, when the vehicle encounters an abnormal situation, a human operator can take over using HMI haptic feedback to feel the road conditions and obstacles, ensuring safe navigation and improving response times and overall safety (Kuru, 2021). Additionally, HITL telemanipulation of unmanned aerial vehicles (UAVs) allows operators to control drones remotely while receiving haptic feedback about the drone’s interactions with its environment. For instance, an operator uses a haptic interface to control the UAV. The haptic feedback provides sensations of wind resistance, surface textures, and physical interactions with obstacles (Zhang et al., 2021). Integrating haptic feedback into HITL operations enhances human perception by providing a multisensory experience. This integration improves the realism of virtual environments and the accuracy and efficiency of tasks, requiring fine motor skills and precise control. Haptic feedback bridges the gap between the virtual and physical worlds, allowing users to interact with digital systems more naturally and intuitively. Thus, the concept of human–machine symbiosis refers to the synergistic relationship between humans and machines, where both entities work together to achieve common goals. This cooperation ensures that the unique strengths of humans (such as intuition, creativity, and decision-making) complement the capabilities of machines (such as speed, accuracy, and endurance). By creating environments where humans and machines work together, the overall system performance can be enhanced. This human–machine symbiosis ensures that both parties contribute to the task, leading to increased productivity and innovation. The key components of behavioral layer are summarized in Figure 4.

System designers, who design and develop new generations of CPSoSs, have to understand and realize which features differentiate them from the traditional CPSs. One of these features is the HITL mechanism that allows CPSoSs to take advantage of some unique human characteristics, making them superior to the machines. The technological assessments are not mature yet to integrate these human-oriented characteristics into machines and robots. As a result, the HITL approach is essential to serve the initial goals of a CPSoS. These characteristics, as have been proposed by the literature (Sowe et al., 2016), are presented below:

• **Cognition.** Humans have a different way of observing a situation than computers do. First, they understand a problem and then make final decisions, even with missing data. Human cognition is the combined result of knowledge, experience, inspiration, and intuition, areas where no current machine can overcome or even approach in some way.

• **Predictability.** Humans are not preordained to perform the same task in the same way every time that they try. This would be a problem in some cases, especially when they have to follow precise instructions. This feature might make them less reliable than a simple computer. However, this unpredictable behavior could be beneficial in a critical situation that has not been distinctly defined in the script of the instructions. The ability of humans to easily adapt to unknown situations makes them a perfect component to provide out-of-the-box solutions in hazardous circumstances.

• **Motivation.** Humans, by their nature, usually require incentives and become more productive when they are assured. Motivation can guide a human to perform more effort on a task than is required. On the other hand, computers and machines follow a particular pipeline of work, and they cannot change the way they perform a task to enhance their productivity.

**FIGURE 5:**
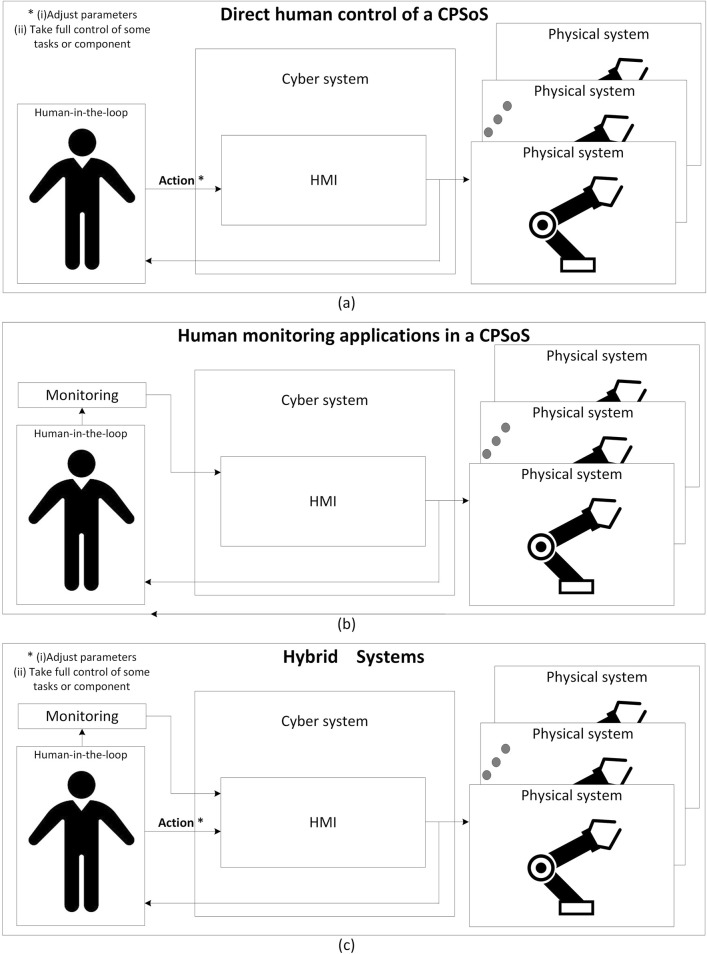
HITL applications with respect to the type of input that humans provide. **(A)**applications in which human plays a leading role of the CPSoS, **(B)**Applications where the system passively monitors humans and then makes decisions for appropriate actions. and **(C)**Hybrid combination of the two types mentioned above.

The HITL applications can be separated into three main categories with respect to the type of input that humans provide:

1. Applications in which the human plays a leading role and directly controls the functionality of the CPSoS as an operator (Figure 5A).
2. Applications where the system passively (Figure 5B) monitors humans (e.g., biometrics, pose) and then makes decisions for appropriate actions.
3. Hybrid combination of the two types mentioned above (Figure 5C).

### 5.1 Direct human control of a CPSoS

The applications in this category can be separated into two different sub-categories related to CPS autonomy. In the first sub-category, operators manage a process close to an autonomous task. The system has complete control of its action, and the user is responsible for adjusting some parameters that may affect the functionality of the system when required for external reasons. An example that can describe a scenario like this is when an operator sets new values to specific parameters on a machine in the industry for changing the operation of the assembly line (e.g., for a new product).

In the second sub-category, the operator plays a more active role in the process by directly controlling several tasks and setting explicit commands for the operation of the machines or robots. An example of this scenario is when an expert operator has to remotely take complete control of a robotic arm for repair purposes.

### 5.2 Human monitoring applications

The applications of this category are represented by systems that passively monitor humans’ actions, behavior, and biometrics. The acquired data can be used to make appropriate decisions. Based on the type of the system reaction, the applications can be separated into two types, namely, open-loop and closed-loop systems.

Open-loop systems continuously monitor humans and visualize (e.g., smart glasses) or send a report with relevant results, which may be helpful or interesting for the operator. The system does not take any further action in this case. The presented results can cover (i) the first level of information, (ii) the second level of information, and (iii) KPIs. First-level information includes measurements that usually are directly received by the sensors (e.g., heart rate, respiratory, and blinking of eyes). After the appropriate process of the first level of information, the second level corresponds to a higher contextual meaning (e.g., drowsiness, awareness, and anxiety level).

Closed-loop systems use the received information from the sensors and the processing results to take action. For example, in an automotive use case, if critical drowsiness of a driver is detected, the car could take complete control of the vehicle or appropriately inform the driver of his/her condition.

#### 5.3 Hybrid systems

In manufacturing, for example, a system monitors the operators’ actions while collaborating with a robot, and it can provide appropriate guided instructions. However, the level of detail of the guided assistant can be modified by the personalized preferences of the user (which may be related to the level of his/her experience, for instance). Hybrid systems take human-centric sensing information as feedback to perform an open or closed-loop action, but additionally, they also take into account the direct human inputs and preferences.

Humans play an essential role in CPSoSs. Their contribution can be summarized into three categories: (i) for data acquisition, (ii) for inference related to their state, and (iii) for the actuation of an action to complete a task of their own or to collaborate with other components of the system (Nunes et al., 2015).

1. Data acquisition:

• Human as an informer. Humans provide the system with information through wearable sensors or other devices/sensors that monitor them.

• Human as a communicator to transfer condensed knowledge. Humans have the special ability to easily understand complicated information, draw conclusions, and transfer filtered, useful, and deductive information to the system.
2. State inference:

• Human as an insider component. Training algorithms and machine learning approaches can be used to recognize the human’s state (e.g., cognitive, physical, emotional, and physiological), which may affect the excellent functionality of a CPSoS or put the user’s safety at risk. When a critical user state is identified, the system can change the typical operation to protect the user or notify them with an appropriate message or warning.

• Human as a feedback component. Based on the state of the users, the system may provide suggestions or recommendations to them. The acceptance of these suggestions by the users can further be utilized by the system as useful feedback, providing more personalized solutions in future similar situations.
3. Actuation:

• Human as actuators. The actions of a human, as a part of a CPSoS, are (i) to set the values of some parameters, (ii) to execute specific tasks, or (iii) to take total control of the system, if required.

**HMI** is referred to as the medium that is utilized for direct communication between humans and machines, facilitating their physical interaction (Ajoudani et al., 2018). In the literature, when there are multi-human users or systems of machines instead of a separate individual machine, HMIs have also been mentioned as cooperative or collaborative HMIs (CHMIs). Nevertheless, for simplicity, they will be referred to as HMIs since the way they function and their primary features remain the same regardless of the number of humans or machines.

Typically, the classical HMI system comprises some standard hardware components, like a screen and keyboard, along with software featuring specialized functionalities, effectively performing as a graphical user interface (GUI). All sensors and wearable devices connected with humans or other components of the CPSoS are also part of an HMI. The HMI has pervasive usage in CPSoSs, allowing each part of the CPSoS to directly interact with a human and vice versa, creating a synergy loop between CPSs and humans. In the future, HMIs will also have social cohesion between humans and machines. Gorecky et al. (2014) suggested that the primary representatives of HMI tools in CPSoSs, which are mainly used for the communication between humans and machines, are automatic speech recognition, gesture recognition, and extended reality, which can be represented by augmented or virtual reality. In such implementations, a touch screen can allow human operators to pass messages to machines. Singh and Mahmoud (2017) presented a framework that is capable of visually acquiring information from HMIs in order to detect and prevent HITL errors in the control rooms of nuclear power plants. The intelligent and adaptable CPSoSs expect the automation systems to be decentralized and support “Plug-and-Produce” features. In this way, the HMIs have to dynamically update and adapt display screens and support elements to facilitate the work of the operators, like IO fields and buttons (Shakil and Zoitl, 2020). Wang et al. (2019a) proposed a graphical HMI mechanism for intelligent and connected in-vehicle systems in order to offer a better experience to automotive users. However, Pedersen et al. (2017) presented a way of connecting an HMI with a software model of an embedded control system and thermodynamic models in a hybrid co-simulation.

Naujoks et al. (2017) presented an HMI for cooperative automated driving. The cooperative perception extends the capabilities of the automated vehicles by performing tactics and strategic maneuvers independently of any driver’s intervention (e.g., avoiding obstacles). The goal of the papers presented by Kharchenko et al. (2014), Orekhov et al. (2016a), and Orekhov et al. (2016b) is to increase the drivers’ awareness through the development and implementation of cooperative HMIs for ITSs based on cloud computing, providing measurements of vehicle and driver’s state in real-time. Johannsen (1997) dealt with HMIs for cooperative supervision and control by different human users (e.g., in control rooms or group meetings). The application in various domains (i.e., cement industry and chemical and power plants) has shown that several persons from different classes (e.g., operators, maintenance personnel, and engineers) need to cooperate.

By integrating haptic technologies into HMIs, human perception and interaction can be significantly enhanced, leading to more effective and efficient HITL operations. Haptic teleoperation allows operators to control remote or virtual systems with the sensation of touch, providing real-time tactile feedback from the remote environment. This feedback increases the operator’s situational awareness and precision by mimicking the sense of touch, thereby enhancing the user’s understanding and control (Cheng et al., 2022). In automotive applications, for instance, haptic feedback in steering wheels or pedals can alert drivers to hazards or guide them through automated driving tasks, thus improving response times and safety (Quintal and Lima, 2021). Additionally, techniques like haptic physical coupling can create a physical connection between the user and the system, enabling more intuitive and direct manipulation of virtual objects or remote devices. This can simulate the weight, texture, and resistance of objects in virtual environments, which is particularly beneficial in training simulations and remote manipulations, where direct human intervention is risky or impossible. For instance, it can be used in simulations for hazardous environments, such as nuclear plants or space missions. Operators can train with realistic tactile feedback, preparing them for real-world scenarios without the associated risks (Laffan et al., 2020). Incorporating haptic feedback into HITL operations can also improve human perception by providing a multisensory experience. This integration not only enhances the realism of virtual environments but also improves the accuracy and efficiency of tasks that require fine motor skills and precise control (Sagaya Aurelia, 2019).

Cooperative HMIs can be used as standalone GUI in aircraft guidance applications, allowing data and other types of graphical information (e.g., routes, route attributes, airspaces, and flight plan tracks). This real-time information enables collaborative decision-making between all associates, such as the crew (the pilot and the copilot), who have to cooperate continuously or interact with air traffic controllers. HMIs can facilitate the users in operational services, such as air traffic flow and capacity management, flight planning, and airspace management. Kraft et al. (2020) and Kraft et al. (2019) investigated the type of information that should be provided to drivers via HMIs in merging or turning left situations to support cooperative driving, facilitating each other’s goal achievement. Cooperation between road users utilizing V2X communication has the potential to make road traffic safer and more efficient (Fank et al., 2021). The exchange of information enables the cooperative orchestration of crucial traffic conditions, like truck overtaking maneuvers on freeways.

In addition to cooperative decision-making facilitated by HMIs, the integration of predictive capabilities and situational awareness is paramount in enhancing the functionality and safety of CPSoSs. **Prediction of operator’s intentions** and **situation awareness** are critical aspects of integrating human capabilities within CPSoSs. By understanding and anticipating human actions and maintaining a high level of awareness, these systems can significantly enhance operational safety, efficiency, and resilience. These concepts further solidify the human–machine symbiosis, ensuring that CPSoSs can effectively adapt to dynamic and unpredictable environments.

**Prediction of operator’s intentions** is a task that can improve the effectiveness of collaboration between CPSoSs and humans. An accurate prediction can be essential, especially in industrial scenarios where the resilience and safety of all CPSoS components mainly depend on the mutual understanding between humans and CPSoSs. So, it seems necessary to design and develop reliable, robust, and accurate human behavior modeling techniques capable of predicting human actions or behavior.

On the one hand, operators are mainly responsible for their safety when they are in the same working environment with a cobot, performing collaborative tasks. However, on the other hand, CPSoSs must have intelligent components that can identify, understand, and even predict operators’ intentions with the primary goal of protecting them from severe injury. A continuous video-capturing component can be used by the prediction system to detect, track, and recognize human gestures or postures, and an artificial intelligence component can be used to predict human intentions. The system can anticipate when unexpected human operations have been detected, or specific human activity patterns have been predicted (Zanchettin et al., 2019). Meanwhile, the cobot can perform other tasks (Garcia et al., 2019). In the literature, a lot of different approaches have been presented to solve the problem of the prediction operator’s intentions, such as a framework for the prediction of human intentions from RGBD data (Casalino et al., 2018). A sparse Bayesian learning-based human intention predictor is used to predict the future human desired position (Li et al., 2019). A temporal CNN with a convolution operator is applied for human trajectory prediction (Zhao and Oh, 2021). A system that detects human intentions through a recursive Bayesian classifier is used, exploiting head and hand tracking data (Casalino et al., 2018). Another human intention inference system that uses an expectation-maximization algorithm with online model learning is employed (Ravichandar and Dani, 2017).

**Awareness:** Situational awareness in HMIs is used to describe the level of awareness that operators/drivers/users have of the situation in order to perform tasks successfully (Endsley, 1995). Based on the definition provided by Vidulich et al. (1994), situational awareness needs to include four specific requirements:

1. to easily receive information from the environment.
2. to integrate this information with relevant internal knowledge, creating a mental model of the current situation.
3. to use this model to direct further perceptual exploration in a continual perceptual cycle.
4. to anticipate future events.

Taking these four requirements into account, situational awareness is defined as the continuous extraction of environmental information, integrating this information with previous knowledge to form a coherent mental picture and using that picture in directing perception and anticipating future events. The system will be able to monitor and understand the user’s state (e.g., fatigue and cognitive level) to produce personalized alarms, warnings, information, and suggestions to the users. A situational awareness application could also provide

• Information streams regarding the task underway, improving focus.

• Personalized reminders regarding other parallel or scheduled tasks significantly improve response time.

• Notifications and visual aids regarding imminent dangers or accident-related factors.

• Environmental values and real-time measurements of sensors.

• KPIs visualizing the effectiveness of the CPSoS functionality.

Situation awareness is essential in cases where a user must intervene in operations and co-operations with highly automated systems in order to correct failed autonomous decisions in CPSoSs (Horváth, 2020). It is also an effective method to keep the mechanical parts of a system and its operators secure and safe; it can be classified into two groups, human and computer awareness (Yang et al., 2018). Moreover, situational awareness for security reasons is critical since it can be used to inform the user about a cyber-attack that takes place in real time (Joo et al., 2018).

## 6 Role of digital twins in optimizing CPSoS ecosystems

Building upon the previously described perception and behavioral layers of the CPSoS general architecture, DTs emerge as a crucial technology that realizes and optimizes this framework. The perception layer’s role in enhancing situational awareness through object detection, cooperative scene analysis, and effective path planning and the behavioral layer’s focus on integrating human operators and supporting HITL interactions are both significantly augmented by the capabilities of DTs. DTs provide a dynamic and real-time simulation of physical systems, creating accurate virtual replicas that enable continuous monitoring and data integration across both layers. More specifically, DTs in the perception layer offer a comprehensive view of the environment by synchronizing data from various sensors and subsystems, ensuring more precise and reliable situational awareness. This integration allows for improved responsiveness to environmental changes and anomalies, enhancing the autonomy and reliability of CPSoSs. In the behavioral layer, DTs facilitate seamless human-machine interfaces by delivering real-time feedback and predictive insights. This supports the HITL approach by allowing human operators to interact with the system using real-time simulations and up-to-date information. Advanced HMIs such as gesture recognition and eXtended Reality technologies are further empowered by DTs, making interactions more intuitive and efficient. Moreover, the predictive capabilities of DTs help anticipate operator intentions, improving the collaborative efforts between humans and the system, especially in critical scenarios where safety and efficiency are paramount.

As such, by integrating DT frameworks into the proposed two-layered architecture, the CPSoS ecosystem is not only optimized but also becomes more resilient and adaptive to the complex demands of various domains, including automotive, industrial manufacturing, and smart cities. This synergy between DTs and the CPSoS architecture leads to smarter, more efficient systems capable of addressing modern challenges with greater efficiency. In the following sections, we will present how DTs can be employed to realize indicative large-scale CPSoSs like smart cities, transportation systems, and aerial traffic monitoring.

### 6.1 Digital twins in prominent examples of CPSoSs

DTs will play a pivotal role in optimizing large CPSoSs by creating virtual replicas of physical systems, allowing for real-time monitoring, simulation, and predictive maintenance. This capability is particularly important for large CPSoSs, where the integration of numerous interconnected subsystems demands precise coordination and management. DTs enhance the functionality and efficiency of these complex systems by providing a unified platform for data integration, analysis, and visualization. By enabling continuous feedback loops between the physical and digital realms, DTs improve decision-making processes, enhance system reliability, and optimize operational performance across diverse domains, including but not limited to smart cities, intelligent transportation systems, and aerial traffic monitoring (Mylonas et al., 2021; Kušić et al., 2023; Wang et al., 2021).

#### 6.1.1 DTs and smart cities

The management and development of smart cities can be revolutionized by DTs as they provide detailed digital replicas of urban environments. These digital models integrate various data sources to deliver real-time insights and simulations, enhancing urban planning, infrastructure maintenance, and environmental monitoring. More specifically, DTs enable city planners to test different scenarios and make data-driven decisions, optimizing the layout and functionality of urban spaces. By modeling traffic flows, optimizing traffic light timings, and reducing congestion, DTs improve urban mobility and air quality. For example, DTs can analyze data from traffic cameras, sensors, and GPS to provide real-time traffic management solutions, facilitating efficient and sustainable urban traffic control (Schwarz and Wang, 2022). Furthermore, DTs allow for the detailed simulation of urban infrastructure, helping planners optimize the layout and design of utilities such as water, electricity, and waste management systems. By providing a virtual model of the city’s infrastructure, DTs enable predictive maintenance and efficient resource allocation, reducing operational costs and improving service delivery (Broo and Schooling, 2023). Additionally, DTs play a crucial role in enhancing public safety and emergency response (Aluvalu et al., 2023). By integrating data from surveillance systems, emergency services, and environmental sensors, DTs provide real-time situational awareness, enabling faster and more coordinated responses to emergencies. For instance, DTs can simulate natural disaster scenarios and help plan effective response strategies, thereby improving the efficiency and effectiveness of emergency management. Another important aspect is that DTs support smart building management by monitoring energy consumption, predicting maintenance needs, and improving overall building efficiency. By providing a virtual model of buildings, DTs help optimize heating, ventilation, and air conditioning (HVAC) systems, lighting, and other building services, contributing to energy savings and improved occupant comfort (Eneyew et al., 2022). Finally, DTs may be utilized for environmental monitoring by integrating data from air quality sensors, weather stations, and other environmental monitoring tools (Purcell et al., 2023). They provide real-time insights into environmental conditions, helping cities monitor pollution levels, manage natural resources, and implement sustainability initiatives. For instance, DTs can help track and manage water quality in urban water systems, ensuring safe and clean water for residents. Overall, DTs enhance the functionality and efficiency of smart cities by providing a unified platform for data integration, analysis, and visualization. This technology enables cities to become more resilient, sustainable, and responsive to the needs of their residents.

#### 6.1.2 DTs and intelligent transportation systems

The complexity of modern transportation systems necessitates sustainable technological innovations. DT technology, as an innovative architecture, is well-suited to examine the lifecycle of various systems in a digital format. DTs support numerous aspects of transportation infrastructure, including transport system monitoring, energy management, traffic forecasting, EV energy consumption forecasting, subway regenerative braking energy forecasting, parking lot management, driver behavior analysis, pedestrian behavior investigation, health system control, and cyber–physical attack detection (Jafari et al., 2023). By enhancing traffic forecasting accuracy through real-time data collection and high-quality models, DTs improve traffic planning and management. This enhancement results in time and cost savings, reduced energy consumption, improved driver wellbeing, and overall better performance. By organizing data and algorithms, DTs can support sustainable urban traffic formation and efficient control (Jiang et al., 2022). For example, they optimize traffic light timings and provide accurate traffic information, facilitating optimal traffic management and extensive EV traffic planning (Kušić et al., 2023; Chomiak-Orsa et al., 2023). Furthermore, DTs are instrumental in predicting and optimizing the energy consumption and production patterns of electrical transportation systems. They enhance the management and optimization of energy consumption, thus improving the operation and performance of these systems (Ketzler et al., 2020; Bhatti et al., 2021). DTs are crucial for the development and operation of autonomous vehicles (Almeaibed et al., 2021). They simulate vehicle behavior under various conditions, allowing for extensive testing and optimization without the risks associated with real-world testing. DTs help in refining algorithms for navigation, obstacle detection, and collision avoidance, making autonomous vehicles safer and more reliable. By providing a comprehensive digital environment, DTs enable the testing of autonomous vehicles in a wide range of scenarios, including adverse weather conditions, complex urban environments, and interactions with other vehicles and pedestrians. This capability is essential for improving the robustness and safety of autonomous driving systems (Bhatti et al., 2021). Another notable application of DT technology is in analyzing and investigating real-time driver and pedestrian behavior, enhancing security and environmental sustainability. By assembling real-time data from drivers and vehicles, DTs transfer crucial information to the physical world, addressing security concerns and promoting sustainability (Yan et al., 2022). Furthermore, DTs play a vital role in detecting cyber and physical attacks in transportation systems, increasingly targeted by hackers due to the integration of wireless and IoT technologies. This capability ensures a secure and reliable environment for all transportation agents (Liu et al., 2020; Damjanovic-Behrendt, 2018). As transportation systems evolve with advanced technologies, they become more vulnerable to cyber and physical attacks. DTs enable the dynamic analysis of transportation systems, providing real-time detection of such attacks. This functionality helps construct a secure and reliable environment for transportation systems, ensuring the safety of all users, including pedestrians (Almeaibed et al., 2021).

#### 6.1.3 DTs and aerial traffic monitoring

DTs are increasingly recognized for their transformative potential in managing aerial traffic, particularly for UAVs and drones. DTs provide a real-time digital replica of physical assets, enabling precise navigation, real-time monitoring, and effective management of aerial operations. This technology is crucial for ensuring the safety, efficiency, and reliability of aerial traffic, especially in urban environments where the density of aerial traffic is high (Wang et al., 2021). In more detail, DTs can enable real-time monitoring of UAVs and drones by providing a comprehensive digital model that mirrors the physical asset. This model integrates data from various sensors, including GPS, cameras, and environmental sensors, to offer a holistic view of the status and environment of UAVs. These real-time data allow for precise navigation, collision avoidance, and efficient route planning, ensuring safe operations even in complex and dynamic environments (Glaessgen and Stargel, 2012; Soliman et al., 2023). DTs also play a critical role in airspace management by providing a comprehensive and integrated view of all aerial activities within a given area. They can simulate different flight scenarios, optimize airspace usage, and manage the traffic flow to prevent collisions (He et al., 2019). DTs support the coordination of multiple UAVs, ensuring that flight paths are optimized and comply with airspace regulations. This capability is particularly important in urban areas where multiple UAVs may be operating simultaneously (Tang et al., 2023; Lv et al., 2021). In emergency response scenarios, DTs are invaluable for enhancing situational awareness and coordination among various entities. For instance, during a natural disaster, DTs can be used to deploy UAVs for search and rescue operations, assess damage, and deliver essential supplies. The real-time data provided by DTs help emergency responders make informed decisions quickly, thereby improving the efficiency and effectiveness of the response (Piperigkos et al., 2023; Wen et al., 2024; Ariyachandra and Wedawatta, 2023).

### 6.2 Digital twins-based integration of smaller CPSoSs into larger CPSoSs

In the previous examples, DTs have been exploited not only to model and optimize the performance of individual CPSoSs like autonomous ground and aerial vehicles but also to integrate these smaller CPSoSs into larger CPSoS ecosystems, like smart city environments. This integration features great potential for creating a synergistic ecosystem where various systems work together seamlessly to enhance overall functionality, efficiency, and resilience. For example, the integration of autonomous ground vehicles into the infrastructure of smart cities exemplifies the convergence of smaller CPSoSs into larger CPSoSs. Autonomous vehicles operate as part of a larger network, interacting with smart traffic management systems, connected infrastructure, and other smart devices to optimize urban mobility. Autonomous vehicles communicate with traffic lights, road sensors, and central management systems to navigate efficiently, reduce traffic congestion, and enhance road safety. This level of integration allows for real-time data sharing and coordinated decision-making, significantly improving the performance of urban transportation systems (Piperigkos et al., 2023; Huang et al., 2024; Hu et al, 2024). Furthermore, a key advantage of integrating smaller CPSoSs (e.g., autonomous vehicles) into larger CPSoSs (e.g., smart cities) is the seamless data integration and management it facilitates. Data from various sources, such as vehicles, smart buildings, environmental sensors, and public transportation systems, can be collected, analyzed, and utilized in a unified platform. This integrated data ecosystem enables better decision-making and proactive management of urban systems. For example, data from autonomous vehicles can be combined with environmental data to monitor and manage urban air quality more effectively (Kopelias et al., 2020; Bayat et al., 2017). In general, the urban ecosystem can be enhanced by enabling real-time data sharing and collaborative decision-making across various systems. This interconnected network of CPSoSs facilitates efficient resource management, improves public services, and increases resilience against disruptions. Integrating transportation systems, energy grids, and public safety networks through DTs allows for optimized urban operations, reduced response times in emergencies, and an overall enhancement in the quality of life for residents (Lv et al., 2022). In the context of urban transportation, multiple CPSoSs can collaboratively work to streamline traffic flow, reduce congestion, and lower emissions by sharing real-time data and predictive analytics (Liu et al., 2023). This synergy allows for dynamic adjustment of traffic signals, real-time rerouting of vehicles, and efficient public transport scheduling. Energy grids can interact with transportation systems to manage the charging of electric vehicles, ensuring that the energy supply meets demand without overloading the grid (Bhatti et al., 2021). This holistic integration supports disaster management, improves emergency response times, and enhances overall urban resilience.

## 7 Challenges and open research issues

The advancement of CPSoSs involves overcoming technical and integration challenges in areas such as cooperative object detection and fusion, cooperative localization and path planning, cooperative SLAM, and HITL integration. These areas are crucial for enhancing the functionality, reliability, and efficiency of CPSoSs. Cooperative object detection and fusion integrate data from various sensors, improving situational awareness in the perception layer. Cooperative localization and path planning optimize navigation and traffic management, aligning with the perception layer’s goals. Cooperative SLAM enables accurate environmental mapping. HITL integration enhances decision-making, linking to the behavioral layer’s focus on human interaction and control. Addressing these challenges is essential for improving performance, safety, and adaptability in diverse and dynamic environments, driving significant advancements in the development and deployment of CPSoSs across various sectors.

### 7.1 Cooperative object detection and fusion

Integrating data from heterogeneous sensors, such as cameras, LiDAR, and radar, remains complex due to differences in data formats, resolutions, and sampling rates, necessitating the development of robust fusion algorithms. Furthermore, developing methods for multi-modal object representation that reconcile discrepancies in perception across sensors is crucial for cohesive and accurate environmental understanding (Arnold et al., 2020; Guo et al., 2021). Managing uncertainties in sensor measurements and fusion processes is vital to enhance the reliability of object detection. Establishing standardized benchmarks and metrics for evaluation is necessary to effectively assess and compare the performance of cooperative object detection systems. Optimizing computational efficiency and energy consumption of algorithms while maintaining high accuracy poses additional challenges. Lastly, understanding human interactions with autonomous systems equipped with cooperative object detection capabilities is essential for ensuring safe integration into mixed-traffic environments.

### 7.2 Cooperative localization and path planning

Cooperative localization for connected CPSs is advancing, but several key research challenges remain. Algorithms need to maintain accurate positioning even when communication is disrupted or networks are patchy. Integrating data from GPS, LiDAR, and cameras is crucial for precise localization. Algorithms must also work well in GPS-unavailable areas. Efficiently processing large data on limited computing power is essential for inter-vehicle communication. Urban areas present signal issues that need advanced processing techniques. Finally, creating realistic tests and benchmarks is vital for ensuring system reliability.

Cooperative path planning involves multiple agents working together to navigate from their respective starting points to designated endpoints. Despite significant advancements, several open issues remain in this domain. For application to real conditions, scalability and efficient coordination require algorithms capable of managing large fleets of CPSs (Halder et al., 2020; Wang et al., 2019b) without compromising performance opting for real-time decision-making for a swift response to dynamic environments (Viana and Aouf, 2018), such as sudden obstacles (Viana et al., 2019). Robust algorithms are needed to maintain path planning when inter-vehicle communication is unreliable. Effectively integrating heterogeneous sensor data from LiDAR, cameras, and GPS can improve decision-making accuracy, but it also requires conflict resolution mechanisms. Furthermore, adapting path planning algorithms to diverse conditions and regulatory environments requires the definition and introduction of constraints. Real-world testing and validation are essential to validate algorithms under diverse conditions, ensuring they meet the stringent requirements of safe and efficient behavior of CPSoSs.

### 7.3 Cooperative SLAM

As mentioned by Saeedi et al. (2016), cooperative SLAM has to face quite some challenges in order to fully exploit the potential of collaboration.

#### 7.3.1 Data distribution

Further investigation is needed to determine which processing architecture, either centralized or distributed, is more efficient for the cooperative fusion of different SLAM solutions. The optimal choice is closely related to the three other major challenges.

#### 7.3.2 Relative poses of robots

The map provided by each robot in its own reference coordinates is called the local map. Each robot aims to integrate all individual local maps to generate a global map of the environment. However, this difficult task requires *a priori* unknown transformation matrices, which relate these maps to one another. The problem of the relative pose of the robot is coupled with the multiple-robot data association problem. Knowledge of one makes the other a simple problem.

#### 7.3.3 Updating maps and poses

Once the relative transformation is determined, the fusion of local maps is necessary. The resulting map should integrate all the information contained within local maps. As a result of updating the maps, the poses of the robots should also be updated. This requires considering the current full trajectory of the robots and new information received from other maps.

#### 7.3.4 Communication requirements

The availability of a medium for data sharing among robots is an important requirement in multiple-robot SLAM. Information between robots can be exchanged via communication channels. The quality of the communication channels is dependent on the (harsh or not) environment. Additionally, the amount of data that needs to be exchanged may have a significant impact on the efficiency of communication. For instance, a local map of thousands of 3D points that needs to be transmitted to a group of robots is not a trivial task. Therefore, rich communication resources are also needed in order to realize cooperative SLAM.

### 7.4 Human in the loop in CPSoSs

In CPSoSs, integrating human feedback into the control loop of a system allows humans to interact with and influence automated processes in real time. However, a series of challenges appear with respect to this component that CPSoSs have to address.

#### 7.4.1 Processing in real time

The complexity of CPSoSs, consisting of a variety of different components, leads to the instantaneous production of a huge amount of data. The processing of these data and real-time decision-making are challenging tasks, considering that human safety is the most important issue. The processing of data in batches could be a solution to this challenge. However, this approach is not reliable in critical situations within CPSoSs, where vital and accurate decisions have to be made quickly to protect human life and security. In other words, the real-time data processing framework requires the system to handle large amounts of data with very low latency while maintaining relatively high performance.

#### 7.4.2 Online streaming of data

CPSoSs are systems of systems that are interconnected, collaborating, and transferring in real-time helpful information and data. Online streaming also requires real-time data processing. In the case of online streaming, the challenge originates from data transfer in an ordered sequence of instances that can usually be accessed once or a few times due to limited computing and storage capabilities. The tremendous growth of data demands switching from traditional data processing solutions to systems that can process a continuous stream of real-time data.

#### 7.4.3 High-dimensional data

High-dimensional data are becoming a prevalent issue in many real-world applications of CPSoSs. The processing of high-dimensional data acquired by different sensors and devices presents a fundamental challenge, leading to more sophisticated methods being developed. High-frequency data refer to data that usually appear as time series, with their values updating very rapidly (i.e., new observations take place every milliseconds-second). The appropriate management of high-frequency data is essential for contemporary CPSoSs. Processing these data introduces new challenges to decision-making tasks, especially when a human takes part in the CPSoS as a HITL component.

#### 7.4.4 Unsupervised learning in data of CPSoSs

Unsupervised learning is a type of learning that tries to discover hidden patterns in untagged data autonomously. This can be beneficially used in real-time applications where the observed data possess a large variety of classes compared to those in a restricted dataset. However, applying this in CPSoSs is a very challenging task as they require accurate and precise results, and usually, there are no “ground truth” data for the evaluation of the method’s accuracy (Ma et al., 2018).

## 8 Lessons learned

In the connected CPSoS, each node perceives the environment and generates data streams shared among all connected nodes, facilitating collaborative perception, localization, and path planning. This interconnectedness allows each node to access a wealth of information about the common environment that would otherwise be unavailable. In practice, the sensing range of each individual CPS is extended according to the sensing capabilities of the other interconnected CPSs (Piperigkos et al., 2021), thus leading to complementary data fusion. The integration of cooperative perception, localization, SLAM, path planning, and HITL forms the foundation of distributed intelligence in CPSoSs. The integration of various sensors across nodes significantly enhances cooperative situational awareness, and the benefits of that type of collaboration have been quantified both theoretically and algorithmically. For example, Fisher information matrix-based analysis (Buehrer et al., 2018) provides indicative insights about the fundamental nature of collaboration and the scaling with network size, anchor placement, neighbor selection, etc., demonstrating how the integration of sensors facilitates swarm navigation in Mars exploration missions (Zhang et al., 2020). Therefore, this interconnected data sharing results in a more comprehensive understanding of the environment, leading to improved decision-making capabilities. For instance, in autonomous driving, shared data from multiple vehicles can provide a clearer picture of road conditions and traffic patterns, enhancing safety and efficiency (Arvanitis et al., 2023). Cooperative localization enables precise positioning even in challenging environments where traditional GPS signals may be unreliable (Piperigkos et al., 2023).

By sharing location data among nodes, CPSoSs can achieve more accurate and reliable navigation. This is crucial for applications such as autonomous vehicles and drones, which rely on precise localization for safe operation. Cooperative SLAM extends the capabilities of individual systems by allowing multiple nodes to collaboratively map their environment; as stated by Saeedi et al. (2016), continuously updating and sharing maps among nodes enhances the overall system’s adaptability to dynamic environments. However, this fact also emphasizes the need for more accurate and up-to-date maps, which are essential for navigation and obstacle avoidance. In the same context, incorporating human feedback into CPSoS operations could potentially enhance system performance in dynamic situations where algorithms might lack situational awareness or adaptability (Alsamhi et al., 2024). This is particularly important in scenarios where automated systems face uncertain or complex situations. HITL systems ensure that human operators can intervene when necessary, providing a safety net for critical operations.

It is expected that the collective intelligence of connected CPSoS nodes will lead to more robust and resilient systems. By distributing computational tasks and decision-making processes across multiple nodes, CPSoSs will be able to handle larger and more complex tasks with greater efficiency. In this distributed type of approach, system scalability can also be enhanced, making it easier to expand CPSoSs to accommodate more nodes and diverse sensors. In the same context, DTs will play a crucial role in optimizing CPSoS ecosystems by creating virtual replicas of physical systems (i.e., smart cities, transportation, etc.), enabling real-time monitoring, predictive maintenance, and scenario simulation. In practice, DTs are expected to enhance the interaction between smaller and larger CPSoSs, facilitating efficient data sharing and collaborative decision-making across various systems, with a direct impact on the quality of life (Lv et al., 2022). Despite significant advancements, several challenges remain, including managing high-dimensional data, ensuring real-time processing, and developing robust algorithms for data fusion and localization. Future research should focus on addressing these challenges to further enhance the capabilities of CPSoSs. Overall, the lessons learned from the development and implementation of CPSoSs highlight the importance of collaboration, data sharing, and human integration in creating intelligent, adaptive, and efficient systems. These insights provide a roadmap for future research and development, aiming to optimize CPSoSs for various applications, from smart cities to autonomous transportation.

## 9 Discussion

In this survey, we examine CPSoSs and their components that improve situational awareness for users, an aspect not thoroughly discussed in previous review papers. By focusing on human integration into CPSs, we include the HITL element and HMI in the CPSoS concept. We also emphasize the crucial role of DTs in optimizing CPSoS ecosystems. The key contributions of this paper consist of an extensive review of current leading practices in connected CPSs and an analysis of a dual-layer architecture with a perception layer for situational awareness and a behavioral layer for incorporating human operators through HITL mechanisms and sophisticated HMI technologies. Furthermore, we provide various datasets and data sources accessible to the research community, concentrating on perception algorithms for scene understanding, localization, mapping, and path planning, along with decision-making and HITL control. We also discuss the incorporation of DTs into CPSoSs, showcasing their applications in smart cities, intelligent transportation systems, and aerial traffic monitoring.

In more detail, this survey offers a comprehensive exploration of the architectural and operational intricacies of CPSoSs, highlighting the dual-layer architecture comprising the perception and behavioral layers. This approach distinguishes our study by providing a detailed examination of how these layers enhance situational awareness and integrate human elements within CPSoSs. The perception layer is meticulously designed to focus on advanced perception algorithms essential for object detection, scene analysis, cooperative localization, and path planning. These capabilities are critical for achieving higher autonomy and reliability in CPSoSs. Unlike other studies that may address these components in isolation, our research integrates these elements into a cohesive framework, demonstrating how they collectively contribute to the overall functionality and efficiency of CPSoSs. The behavioral layer emphasizes the integration of human operators through HITL mechanisms and advanced HMI technologies. This layer underscores the importance of human cognition, adaptability, and motivation, which are crucial for operational excellence in CPSoSs. Our study uniquely addresses the challenges and benefits of incorporating human feedback and interaction into automated systems, promoting a human–machine symbiosis that enhances decision-making and system flexibility. Additionally, we delve into the role of DTs in optimizing CPSoS ecosystems. Our research highlights the potential of DTs in smart cities, intelligent transportation systems, and aerial traffic monitoring, showcasing how they facilitate real-time data integration, predictive maintenance, and improved decision-making. The discussion extends to the integration of smaller CPSoS into larger systems, emphasizing that seamless communication and coordination among subsystems enhance overall system performance and urban management.

Our research provides valuable insights into how evolving CPSoSs can transform collaborative and collective decision-making and significantly impact various industries and disciplines. The integration of HITL mechanisms and HMIs enhances real-time interaction and feedback between humans and machines, leading to more informed and effective decision-making processes. For example, in industrial production, the ability to integrate human expertise with automated processes can create more flexible and adaptive manufacturing systems. Operators can provide real-time input and adjustments, ensuring that production lines can quickly respond to changes in demand or unforeseen issues, thus improving efficiency and reducing downtime. In the context of smart cities, CPSoSs can enhance urban mobility, energy management, and public safety. For instance, the integration of autonomous ground vehicles within smart city infrastructure allows for optimized traffic flow and reduced congestion through real-time data sharing and coordinated decision-making. This not only improves the efficiency of transportation systems but also contributes to environmental sustainability by reducing emissions. The research community can greatly benefit from this manuscript by leveraging the frameworks and methodologies presented to establish robust CPSoSs. Our detailed discussion on perception and behavioral layers, as well as the integration of DTs, offers a comprehensive guide for developing and optimizing CPSoSs. By addressing both technical and human-centric aspects, our study provides a holistic approach to understanding and implementing CPSoSs. Researchers can build on these insights to explore new opportunities, address current challenges, and develop more resilient and adaptable systems that meet the evolving demands of various industries. Overall, this survey emphasizes the transformative potential of CPSoSs in enhancing collaborative and collective decision-making, operational efficiency, and quality of life across different sectors. The insights gained from this study serve as a valuable resource for the research community, guiding future innovations and advancements in the field of CPSoSs.

### 9.1 Future directions

As CPSoSs continue to evolve, several key areas require further attention and development to ensure their effective implementation and optimization. One critical area is security and trust. Since CPSoSs are tightly integrated with human elements and involve diverse, interconnected systems, ensuring robust security measures is paramount. Traditional security measures often fall short due to the complexity, autonomy, and heterogeneity of CPSoSs, as well as the presence of legacy components. Therefore, a new security and trust mechanism tailored for CPSoSs is essential. This mechanism must be embedded from the design phase and continuously updated throughout the system’s lifecycle to adapt to emerging cybersecurity threats. Implementing a security-by-design principle ensures that security components can accommodate varying security needs and performance capabilities across different CPSs. Additionally, specialized security monitoring tools should be integrated to detect, respond to, and mitigate security breaches in real time, ensuring resilience even under unforeseen conditions. Another promising direction is the development of systems based on the human-agent–robot teamwork (HART) framework (Demir et al., 2020). This framework emphasizes collaborative interactions between humans and fully or semi-autonomous machines, aiming to harness hybrid intelligence—the combined strengths of human intuition and machine precision. Such synergy can significantly enhance productivity, innovation, and overall system performance. Future research should focus on creating environments that facilitate seamless co-working between humans and machines, optimizing both human cognitive abilities and machine efficiency. This cooperative model not only enhances operational effectiveness but also promotes adaptability in response to dynamic and complex environments, leading to more resilient and innovative CPSoS (Verhagen et al., 2022). By addressing these future directions, the field of CPSoSs can advance toward more secure, efficient, and human-centric systems, ensuring their relevance and effectiveness in various domains.

Finally, Table 10 summarizes the main lessons learned about the currently matured areas that we presented previously, as well as the corresponding current and future challenges derived from the previous discussion.

**TABLE 10:** Summary of mature, current, and future challenges in CPSoS areas of interest.

Area of interest	SOTA (Mature)	Current challenges	Future challenges
Cooperative object detection and fusion	Data fusion from multiple sensors improves situational awareness and enhances decision-making capabilities	Fusion of heterogeneous data to address discrepancies in data formats and resolutions	Robust algorithms to ensure real-time, multi-modal perception in dynamic environments
Cooperative localization and path planning	Cooperative localization improves precision in GPS-denied environment	Ensuring accurate positioning and path planning under network delay	Scalable algorithms for large CPS fleets with minimal latency and error in real-time localization and path palling
Cooperative SLAM	Cooperative SLAM enables real-time environment mapping	Managing data fusion for distributed SLAM and ensuring up-to-date maps in dynamic environments	Real-time, large-scale SLAM algorithms that are robust to communication latency and noise
HITL systems	HITL ensures human intervention in critical decision-making scenarios, improving safety	Managing the balance between human input and automation in fast-evolving situations	Seamless AI-human interaction for adaptive, safe, and autonomous decision-making in diverse environments
DTs	DTs enable real-time monitoring and predictive maintenance	Integrating smaller and larger CPSoS with DTs to enhance data sharing and decision-making	Full integration of DTs for autonomous, real-time system optimization across different sectors
Security and trust	Basic security mechanisms embedded in traditional CPSoS systems	Ensuring real-time breach detection and secure communication in interconnected environments	Developing security-by-design frameworks for heterogeneous CPSoSs
HART	Collaborative interactions between humans and semi-autonomous systems enhance productivity	Optimizing hybrid intelligence by combining human intuition with machine precision	Creating resilient environments for seamless human–machine cooperation, leveraging cognitive capabilities

## 10 Conclusion

This survey provides a comprehensive review of current best practices in connected CPSoSs. We present a detailed CPSoS architecture that facilitates collective intelligence through sensor fusion, scene analysis, cooperative localization and mapping, and user state monitoring. By examining all aspects of these areas, we offer insights into HITL-oriented datasets, including face analysis and landmark extraction, pose estimation, hand and gesture recognition, action recognition, and speech analysis. Through this survey, readers can gain a deep understanding of the current status, advancements, and challenges in adopting autonomous CPSs and CPSoSs within a continuously evolving technological landscape. We highlight the importance of integrating HITL mechanisms and HMIs to achieve a CPSS paradigm. Additionally, we emphasize the critical role of DTs in optimizing CPSoS ecosystems, demonstrating their applications in smart cities, intelligent transportation systems, and aerial traffic monitoring. By addressing the dual-layer architecture encompassing the perception and behavioral layers, this survey underscores the necessity of enhancing situational awareness and integrating human expertise into automated processes. The insights provided herein are intended to guide future research and development in creating more resilient, efficient, and intelligent CPSoS, ultimately contributing to improved urban ecosystems and industrial environments.

## Glossary

ADAS: Advanced driver assistance system
AR: Augmented reality
CAV: Connected and automated vehicle
CL: Cooperative localization
CNN: Convolutional neural network
CPS: Cyber–physical system
CPSoS: Cyber–physical system of systems
CPSS: Cyber–physical–social system
DT: Digital Twin
FPFH: Fast point feature histogram
GD: Gradient descent
GNSS: Global navigation satellite system
GPS: Global Positioning System
HITL: Human in the loop
HMI: Human–machine interface
ICP: Iterative closest point
IMU: Inertial measurement unit
IoT: Internet of Things
ITS: Intelligent transportation system
KF: Kalman filter
KPI: Key performance indicator
LiDAR: Light detection and ranging
LO: LiDAR odometry
LOAM: LiDAR odometry and mapping
LS: Least squares
RSS: Received signal strength
SLAM: Simultaneous localization and mapping
SPAWN: Sum–product algorithm over wireless networks
UAV: Unmanned aerial vehicle
UGV: Unmanned ground vehicle
UUV: Unmanned underwater vehicle
V2I: Vehicle-to-infrastructure
V2V: Vehicle-to-vehicle
V2X: Vehicle-to-everything
VANET: Vehicular *ad hoc* network
VO: Visual odometry
XR: eXtended Reality
WSN: Wireless sensor network
